# Recastable assemblies of carbon dots into mechanically robust macroscopic materials

**DOI:** 10.1038/s41467-023-42516-8

**Published:** 2023-10-25

**Authors:** Bowen Sui, Youliang Zhu, Xuemei Jiang, Yifan Wang, Niboqia Zhang, Zhongyuan Lu, Bai Yang, Yunfeng Li

**Affiliations:** State Key Laboratory of Supramolecular Structure and Materials, College of Chemistry, Jilin University, Changchun, 130012 China

**Keywords:** Materials science, Nanoscience and technology

## Abstract

Assembly of nanoparticles into macroscopic materials with mechanical robustness, green processability, and recastable ability is an important and challenging task in materials science and nanotechnology. As an emerging nanoparticle with superior properties, macroscopic materials assembled from carbon dots will inherit their properties and further offer collective properties; however, macroscopic materials assembled from carbon dots solely remain unexplored. Here we report macroscopic films assembled from carbon dots modified by ureido pyrimidinone. These films show tunable fluorescence inherited from carbon dots. More importantly, these films exhibit collective properties including self-healing, re-castability, and superior mechanical properties, with Young’s modulus over 490 MPa and breaking strength over 30 MPa. The macroscopic films maintain original mechanical properties after several cycles of recasting. Through scratch healing and welding experiments, these films show good self-healing properties under mild conditions. Moreover, the molecular dynamics simulation reveals that the interplay of interparticle and intraparticle hydrogen bonding controls mechanical properties of macroscopic films. Notably, these films are processed into diverse shapes by an eco-friendly hydrosetting method. The methodology and results in this work shed light on the exploration of functional macroscopic materials assembled from nanoparticles and will accelerate innovative developments of nanomaterials in practical applications.

## Abstract (web summary)

The assembly of nanoparticles into macroscopic materials forms the basis for various advanced material applications. Here, the authors develop macroscopic materials that are both recastable and mechanically robust, despite being composed purely of carbon dots.

## Introduction

Self-assembly of nanoparticles into macroscopic materials is an important target of materials science and nanotechnology, since it paves an avenue towards innovative materials with functions through collective couplings of the optical, electrical, and mechanical properties of the individual nanoparticle^1–5^. To date, several macroscopic materials, such as gels^6,7^, aerogels^8,9^, glassy materials^10–12^, colloidal liquid crystals^13^, photonic crystals^14^, and superlattices^15–17^, have been constructed through utilizing nanoparticles with diverse sizes, shapes, compositions, and interactions among them^2,18^. These macroscopic materials assembled from nanoparticles provided applications in electronics^19^, biomedicine^20^, energy storage^9^, and highly efficient catalysts^21^. These nanoparticles usually have small ligands or short polymer chains grafted on their surface^22,23^. These short chains cannot lead to enough interchain interactions between neighboring nanoparticles^24^. Therefore, the macroscopic materials assembled from nanoparticles were generally brittle, mechanically weak, and lack of flexibility^1,2^. These limitations hindered the fabrication of robust macroscopic devices from nanoparticles with diverse functionalities^1^. To improve the mechanical properties of macroscopic materials from nanoparticles, the covalent or non-covalent cross-linking interactions were used^14,25^, although these materials were usually not recastable and sustainable. Mass-production of functional nanoparticles and their assembly into macroscopic materials with mechanical robustness, sustainable processing, and recastable ability will be highly desirable in the discovery of a class of transformative materials^1,2^.

Carbon dots (CDs), as emerging nanoparticles, have attracted extensive interests in fundamental and practical perspectives, since they exhibited appealing characteristics, such as low toxicity, good biocompatibility, easy and low-cost mass-production, facile modification, environment-friendly, and superior photoelectric and photoluminescence properties^26–28^. Currently, bottom-up strategies are commonly used to synthesize CDs from small organic molecules or polymers^26–28^. CDs show characteristic core-shell structures with a carbonized core and a shell with abundant functional groups or short polymer chains^26,29^. In particular, the functional groups or polymer chains facilitated the chemical modification of CDs, further advancing their development in the nanocomposites and the functional devices^28,30,31^. Through using CDs as building blocks, nanocomposite materials have been prepared, including nanoassemblies^32–35^, glassy materials^36^, hydrogel^37^, supraparticles^38,39^, nanocomposite polymers^31,40^, and colloidal liquid crystals^41^. These CDs nanocomposites showed appealing properties and had diverse applications in photocatalysis^42^, cancer therapy^32,33^, supercapacitors^43^, sensors^44^, optoelectronic devices^45,46^, laser^47^, and actuators^48^. Notably, the macroscopic materials by using CDs as building blocks will inherit the excellent properties of the CDs and generate collective properties not hosted in the individual CDs^1^, however, the macroscopic materials assembled from CDs solely are not reported. Furthermore, the qualitative or quantitative understanding of interactions that mediate the collective properties of the macroscopic materials assembled from CDs is limited. Without rational chemical modifications, CDs usually showed relatively weak interactions among them, which cannot result in mechanically strong and solvent-stable macroscopic materials assembled from CDs solely. Moreover, in the solution processing of CDs, the interfacial tension resulted from the drying solvent front can lead to tensile stresses that exceed the strength of the CD assemblies, resulting in uncontrollable cracking in macroscopic materials assembled from CDs^1^.

Well-defined supramolecular structures and materials were prepared through non-covalent interactions, such as hydrophobic interactions, electrostatic interactions, hydrogen bonds etc^49^. Among them, hydrogen bonding is especially suitable as the non-covalent interaction for supramolecular synthesis because of the more controllable directionality and saturation of hydrogen bonds^49,50^. In particular, ureido pyrimidinone (UPy) groups enable a quadruple hydrogen bonding moiety with a high dimerization constant^50^. CDs usually have a relatively flexible carbonized core and a shell of short polymer chains with abundant functional groups which can be modified by functional moieties. The CDs modified by UPy groups would self-assemble into macroscopic materials with mechanical robustness and photoluminescence properties. Because these macroscopic materials combined the strong association and reversibility of hydrogen bonds between UPy groups with many attractive features of CDs, such as facile modification, environment-friendly, and photoluminescent properties. Moreover, UPy groups have low absorption of visible light, which prevents them from interfering in the photoluminescence of CDs.

Herein, we report a recastable, self-healing, and mechanically robust macroscopic material self-assembled from CDs modified by UPy groups. We showed that the modification amounts of UPy groups on the nanoscale CDs-UPy were precisely tailored, leading to a regulation of the interaction forces between CDs-UPy. The transparent macroscopic CDs-UPy films preserved the fluorescent properties from the original CDs. More importantly, the macroscopic CDs-UPy films exhibited collective properties, e.g., superior mechanical properties, self-healing, and recastable ability. The Young’s modulus of CDs-UPy film was over 490 MPa. The breaking strength of the CDs-UPy film was over 30 MPa. The CDs-UPy films exhibited a good stability in commonly used organic solvents with the capacity of the solvent resistance and non-swelling behaviors. The CDs-UPy films are recastable with the maintenance of their original mechanical properties after several recasting usages. Through scratch healing and welding experiments, the CDs-UPy films show excellent self-healing properties under mild conditions. Furthermore, the macroscopic films with tunable fluorescence properties were achieved by doping other functional CDs in the CDs-UPy films. The molecular dynamics simulation unveiled that the interplay of the interparticle hydrogen bonding and the intraparticle hydrogen bonding governed the formation and mechanical properties of the CDs-UPy macroscopic films. Notably, we showed that CDs-UPy macroscopic films can be processed into diverse shapes by a facile and environmental-friendly hydrosetting method.

## Results

### Synthesis and characterization of CDs-UPy

**Fig. 1:**
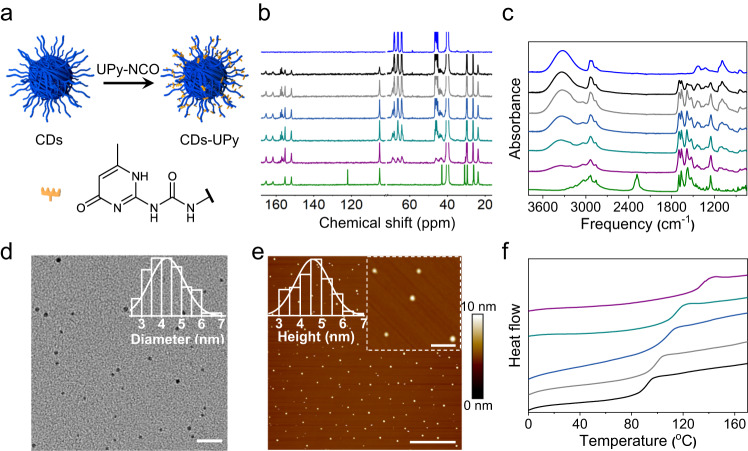
Preparation and characterization of the CDs-UPy. **a** Schematic of the CDs-UPy preparation. **b**, **c** ^13^C NMR (**b**) and FTIR (**c**) spectra of the CDs (blue), UPy-NCO (olive), and CDs-UPy with *α* of 0.89 (black), 1.26 (gray), 1.51 (dark blue), 1.80 (dark cyan), and 2.30 (purple) mmol g^-1^. **d** Representative TEM image of the CDs-UPy with *α* of 2.30 (CDs-2.30UPy). The inset shows the diameter distribution of the CDs-2.30UPy by analyzing 100 CDs-UPy. The scale bar is 20 nm. **e** Representative AFM image of the CDs-UPy with *α* of 2.30 (CDs-2.30UPy). The scale bar is 3 μm. The left inset shows the height distribution of CDs-2.30UPy by analyzing 100 CDs-UPy. The right inset shows the enlarged AFM image of the CDs-2.30UPy. The scale bar is 500 nm. **f** DSC curves of the CDs-UPy with *α* of 0.89 (black), 1.26 (gray), 1.51 (dark blue), 1.80 (dark cyan), and 2.30 (purple) mmol g^-1^. Source data are provided as a Source Data file.

Figure 1a shows the schematic of the preparation of CDs-UPy that self-assemble into macroscopic materials through the hydrogen bonding. To prepare pristine CDs, an aqueous solution of polyvinyl alcohol (PVA) was heated in a sealed Teflon-lined stainless-steel autoclave at 200 °C for 6 h. After cooling to room temperature, CDs with plenty of hydroxyl groups were obtained after purification and lyophilization (Supplementary Fig. 1 and Supplementary Note 1). The CDs-UPy were prepared through the reaction between CDs and 2(6-isocyanatohexylaminocarbonylamino)-6-methyl-4[1H]pyrimidinone (UPy-NCO) catalyzed by dibutyltin dilaurate in the dimethyl sulfoxide (DMSO). The CDs-UPy were purified by multiple cycles of precipitation and washing. Light yellow solids were obtained after lyophilization.

To determine the modification amounts of UPy groups on the CDs-UPy, we performed the elemental analysis of the CDs-UPy (Supplementary Table 1). The modification amounts of UPy groups, *α*, was subsequently calculated by analyzing the content of nitrogen element. In the present work, we explored the structures and properties of macroscopic materials self-assembled from the CDs-UPy by varying *α*, with *α* representing the value of mmol UPy moieties in 1 gram of the CDs-UPy. The *α* values were controlled in the range of 0.89 to 2.30 mmol g^−1^ by tuning the feed amounts of UPy-NCO (Supplementary Table 2).

The successful synthesis of the CDs-UPy was confirmed with ^1^H and ^13^C nuclear magnetic resonance (NMR) and Fourier transform infrared spectroscopy (FTIR) (Supplementary Figs. 2–7, and Fig. 1b, c). The peaks appeared at ≈6, 2, and 1 ppm in the ^1^H NMR spectra of the CDs-UPy, which were assigned to the hydrogen atoms of UPy groups. The peaks appeared at ≈105, 151, 155, 161, and 165 ppm in the ^13^C NMR spectra of the CDs-UPy, which were assigned to the carbon atoms of UPy groups, demonstrating the successful modification of UPy groups on the pristine CDs^51^. Moreover, the appearance of a peak at ≈157 ppm that was assigned to carbon atom of carbamate groups (-NHCOO-), indicated the successful reaction between UPy-NCO and the pristine CDs^52^ (Fig. 1b). The pristine CDs and CDs-UPy showed core-shell structure with UPy motifs grafting on the shell of the pristine CDs, which was characterized by transverse (*T*_2_) relaxation times in NMR measurements^53–55^ (Supplementary Fig. 8 and Supplementary Note 1). The CDs-UPy showed peaks at ≈1698 cm^−1^(C = O stretching of pyrimidinone), ≈1664 cm^−1^(C = O stretching of urea), ≈1585 cm^−1^(C = C stretching), ≈1524 cm^−1^(N-H bending), and ≈1252 cm^−1^(C-N stretching), which are the characteristic FTIR peaks of UPy moieties, indicating the successful grafting of UPy groups on the pristine CDs (Fig. 1c).

Figure 1d shows the representative transmission electron microscopy (TEM) image of the CDs-UPy with *α* of 2.30 mmol g^−1^. The CDs-UPy showed a dot-like shape with an average diameter of 4.3 ± 0.9 nm, which was a bit larger than that of pristine CDs with an average diameter of 2.8 ± 0.5 nm (Supplementary Fig. 1a). The size of the CDs-UPy almost maintained at ≈4.0 nm when *α* changed from 0.89 to 2.30 mmol g^−1^ (Supplementary Fig. 9). Figure 1e shows the representative atomic force microscopy (AFM) image of the CDs-UPy with *α* of 2.30 mmol g^−1^. The average height of the CDs-UPy was 4.6 ± 0.8 nm, which was in agreement with their average diameter determined by TEM. The height of the CDs-UPy remained around ≈4.0 nm when *α* changed from 0.89 to 2.30 mmol g^−1^ (Supplementary Fig. 10). Differential scanning calorimetry (DSC) curves of the CDs-UPy exhibited the glass transition temperature (*T*_g_). The appearance of *T*_g_ was attributed to multiple hydrogen bonding derived from the modification of UPy moieties^49^. As *α* increased from 0.89 to 2.30 mmol g^−1^, the *T*_g_ of the CDs-UPy increased from ≈92 to ≈135 °C (Fig. 1f). Supplementary Figure 11a shows the UV/Vis absorption spectra of the pristine CDs, UPy-NCO, and the CDs-UPy. All the CDs-UPy had absorption peak at ≈283 nm which was the characteristics absorption peak of pyrimidinone in UPy moieties^56^. As *α* varying from 0.89 to 2.30 mmol g^−1^, the absorbance values of CDs-UPy at 283 nm increased, which further indicated the successful preparation of the CDs-UPy with different *α* values. Under the excitation of 370 nm, the photoluminescence (PL) spectra of the pristine CDs and the CDs-UPy with different *α* in DMSO exhibited blue emission with peaks at ≈445 nm (Supplementary Fig. 11b).

### Macroscopic films self-assembled from CDs-UPy

**Fig. 2:**
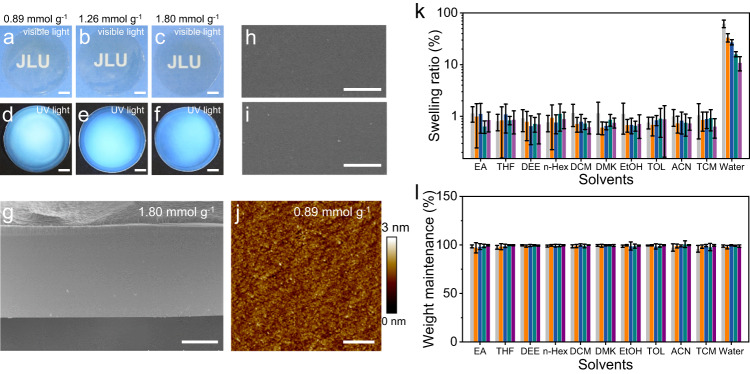
Macroscopic films self-assembled from the CDs-UPy. **a**–**f** Representative photographs of macroscopic films of the CDs-UPy with *α* of 0.89 (**a**, **d**) 1.26 (**b**, **e**), and 1.80 mmol g^−1^ (**c**, **f**). The photographs were taken under the ambient light (**a**–**c**) and the ultraviolet lamp (**d**–**f**). The scale bars are 1 cm. **g** Representative cross-sectional SEM image of the macroscopic film of the CDs-UPy with *α* of 1.80 mmol g^-1^. The scale bar is 50 μm. **h**, **i** Representative SEM images of the upper surface (**h**) and lower surface (**i**) of the macroscopic film with *α* of 1.80 mmol g^-1^. The scale bars are 500 μm. **j** Representative AFM image of the macroscopic film with *α* of 0.89 mmol g^-1^. The scale bar is 400 nm. **k**, **l** Swelling ratios (**k**) and weight maintenances (**l**) of macroscopic CDs-UPy films with *α* of 0.89 (light gray), 1.26 (orange), 1.51 (dark blue), 1.80 (dark cyan), and 2.30 (purple) mmol g^-1^ in different solvents. The error bars represent standard deviations based on three independent measurements. Ethyl acetate (EA), tetrahydrofuran (THF), diethyl ether (DEE), n-hexane (n-Hex), dichloromethane (DCM), acetone (DMK), ethanol (EtOH), toluene (TOL), acetonitrile (ACN), trichloromethane (TCM), and DI water (Water). Source data are provided as a Source Data file.

The macroscopic films self-assembled from the CDs-UPy were prepared by the commonly used solvent-casting method. The CDs-UPy solution in DMSO was first added into a glass petri dish. After the solvent evaporation, the CDs-UPy films were obtained through the interactions of hydrogen bonding among the CDs-UPy. To prove the presence of hydrogen bonding, FTIR measurements at varied temperature from 35 to 155 °C were performed (Supplementary Fig. 12). As the temperature increased, the peaks in the range of 3100–3500 cm^−1^ (N-H and -OH stretching vibration) narrowed, and the peak intensity decreased. The O-H or N-H stretching vibration of the CDs-UPy shifted from ≈3310 to ≈3360 cm^−1^ as temperature increased from 35 to 155 °C. The formation of hydrogen bonds results in the decrease of the stretching vibration frequency and a shift toward lower wavenumber in the FTIR spectra^57–59^. As temperature increased from 35 to 155 °C, the hydrogen bonds in the CDs-UPy films could be partially cleaved or weakened, leading to the increase in the O-H or N-H stretching vibration frequency and a shift toward higher wavenumber in the FTIR spectra^57–59^. These results indicated the presence of hydrogen bonding among the CDs-UPy. The CDs-UPy films with *α* increased from 0.89 to 1.80 mmol g^−1^ can be easily peeled off from the glass petri dish to obtain robust self-standing films (Fig. 2a-f). The CDs-UPy films were flat, transparent, and light yellow. We used the scanning electron microscopy (SEM) to study the morphology of the CDs-UPy film. The film with a thickness of ≈100 μm showed a smooth fracture surface (Fig. 2g and Supplementary Fig. 13) without observable pores. Moreover, the upper surface and lower surface of the CDs-UPy film are free of pores (Fig. 2h,i and Supplementary Fig. 14). Figure 2j showed the AFM image of the macroscopic film (Supplementary Fig. 15). The roughness values, *R*_a_ and *R*_q_, of the film were 0.25 and 0.32 nm, respectively. These morphological characterizations of the CDs-UPy films indicated the homogeneity of the films. The films were transparent with transmittances over 80% in the wavelength range of 450–900 nm (Supplementary Fig. 16a). The films showed blue emission under the excitation of 382 nm which was in agreement with those of the CDs-UPy solutions (Supplementary Fig. 16b). Interestingly, the strategy in this work enabled the combination of the CDs-UPy with other functional CDs to achieve the macroscopic films with tunable properties. For example, the CDs-UPy film doped with red- or green-emissive CDs led to the macroscopic films with a red and green emission at 630 and 495 nm, respectively (Supplementary Fig. 17).

We studied the stability of the CDs-UPy films in commonly used solvents at room temperature. The CDs-UPy films with different *α* values almost showed no swelling behaviors in ten kinds of organic solvents (Fig. 2k). In contrast, the CDs-UPy films swelled in the water. As *α* increased from 0.89 to 2.30, the equilibrium swelling ratio of the CDs-UPy films in water decreased from 62.3 ± 10.4% to 10.9 ± 3.4%. The different swelling behavior of CDs-UPy films in organic solvents and water was attributed to the solvent polarity. The polarity of water is larger than that of organic solvents used in this work. The organic solvents with the small polarity cannot break the strong hydrogen bonding in CDs-UPy films (Supplementary Fig. 18), so the CDs-UPy films showed non-swelling behavior in the organic solvents used in this work. While, water has the ability to form hydrogen bonding with CDs-UPy and can partially break the hydrogen bonding between CDs-UPy (Supplementary Fig. 18). Therefore, the CDs-UPy films swelled in water. We also evaluated the capacity of the solvent resistance of CDs-UPy films by measuring the dry weight of the CDs-UPy films after being immersed in solvents and the water for 24 h at room temperature. The CDs-UPy films maintained their original weights after the treatments in all the solvents (Fig. 2l). DSC curves of the CDs-UPy films also exhibited obvious *T*_g_ which was similar to that of CDs-UPy solids. As *α* increased, the *T*_g_ of the CDs-UPy film increased (Supplementary Fig. 19).

### Mechanical properties of CDs-UPy films

**Fig. 3:**
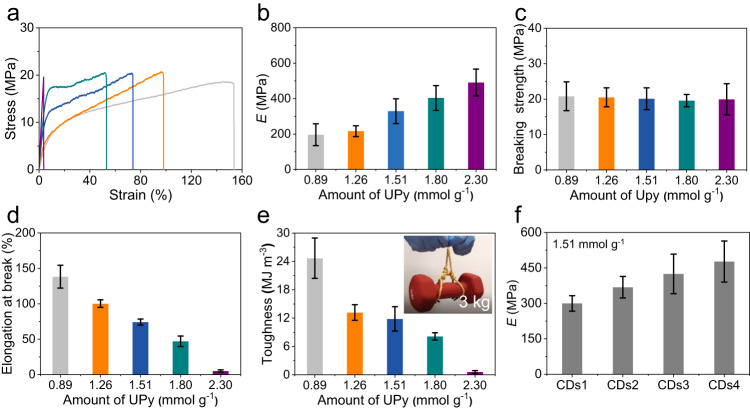
The mechanical properties of the CDs-UPy films. **a**–**e** Representative stress−strain curves (**a**), tensile Young’s modulus, *E* (**b**), breaking strength (**c**), elongation at break (**d**), and toughness (**e**) of CDs-UPy films with *α* of 0.89 (light gray), 1.26 (orange), 1.51 (dark blue), 1.80 (dark cyan), and 2.30 (purple) mmol g^-1^. The inset in **e** showed the photograph of lifting a 3 kg dumbbell by a CDs-1.80UPy film with the thickness of 0.1 mm, length of 5 cm, and width of 1 cm. **f** *E* of CDs-UPy films made from polyvinyl alcohol of different molecular weights with *α* of 1.51 mmol g^-1^. The CDs1, CDs2, CDs3, and CDs4 represent the CDs made from the polyvinyl alcohol with *M*_w_ of 9000-10,000, 31,000-50,000, 89,000-98,000, and 130,000 g mol^−1^, respectively. The error bars represent standard deviations based on at least three independent measurements. Source data are provided as a Source Data file.

In the following experiments, we explored the mechanical properties of CDs-UPy films with different *α* values by tensile measurements. Figure 3a shows the exemplary tensile stress−strain curves of CDs-UPy films with different *α* values. At a small strain, all the stress changed with the strain linearly which was related to the elastic behavior of the CDs-UPy films. The break of the CDs-UPy film with *α* of 2.30 mmol g^−1^ happened in the elastic region with the strain of 5.13 ± 1.75%. With the further increase in the strain, CDs-UPy films with *α* of 0.89, 1.26, 1.51, and 1.80 mmol g^−1^ yielded. Their stress increased with the strain nonlinearly, which was related to the plastic behavior. Further increases in the strain led to the break of CDs-UPy films. Based on the tensile stress−strain curves, we calculated the tensile Young’s modulus of CDs-UPy films, *E*, with varying *α* values (Fig. 3b). Higher *α* values led to the larger values of *E*. The *E* value increased from 196.01 ± 61.36 to 490.48 ± 75.92 MPa as *α* increased from 0.89 to 2.30 mmol g^−1^. The breaking strength of CDs-UPy films showed little variation as a function of *α* values (Fig. 3c), with the value of ≈20 MPa. Moreover, the elongation at break of CDs-UPy films decreased from 138.31 ± 16.14% to 5.13 ± 1.75% as *α* increased from 0.89 to 2.30 mmol g^−1^ (Fig. 3d). Furthermore, as *α* increased from 0.89 to 2.30 mmol g^−1^, the toughness of the CDs-UPy films decreased from 24.68 ± 4.27 to 0.59 ± 0.30 MJ m^−3^ (Fig. 3e). The CDs-1.80UPy film was strong enough to lift a dumbbell of over 50,000 folds of its own weight. As a control experiment, we also functionalized PVA with UPy moieties with *α* of 1.51 mmol g^−1^ (Supplementary Fig. 20a,b and Supplementary Table 1). The breaking strength and elongation at break of PVA-UPy films were similar to those of CDs-UPy films with *α* of 1.51 mmol g^−1^ (Supplementary Fig. 20c,d). The tensile Young’s modulus of CDs-UPy films was ≈40 MPa larger than that of PVA-UPy films (Supplementary Fig. 20e).

To further exploit the energy dissipation and recovery properties of the CDs-UPy films, we performed the cyclic loading−unloading tensile test. For the CDs-UPy films with *α* increasing from 0.89 to 1.80 mmol g^−1^, the cyclic loading−unloading tensile tests were carried out at 20% strain (Supplementary Fig. 21a-d). Since the elongation at break of the CDs-UPy film with *α* of 2.30 mmol g^−1^ was 5.13 ± 1.75%, the cyclic loading−unloading tensile tests of the CDs-UPy film with *α* of 2.30 mmol g^−1^ were performed at 2% strain (Supplementary Fig. 21e). Because the tests were performed in the elastic region, the CDs-UPy films with *α* of 2.30 mmol g^−1^ exhibited no hysteresis at each cycle, indicating that the energy dissipation is not significant. The CDs-UPy films with *α* increasing from 0.89 to 1.80 mmol g^−1^ exhibited obvious hysteresis at each cycle, indicating the existence of energy dissipation. We calculated the energy dissipation of each cycle by integrating the area of hysteresis loops (Supplementary Fig. 22a). For all the films, the energy dissipation decreased as the number of cycles increased. At the first cycle, the dissipated energy increased from 2,041.26 ± 724.96 kJ m^−3^ to 2,743.52 ± 394.01 kJ m^−3^ as *α* increased from 0.89 to 1.80 mmol g^−1^. After the first cycle, the dissipated energy of CDs-UPy films at each cycle was similar. Furthermore, we evaluated the recovery ability of the CDs-UPy films by calculating the ratios of the dissipated energy in each cycle to that of the first cycle. The recovery ratio of the CDs-UPy films with varying *α* values decreased as the number of cycles increased (Supplementary Fig. 22b). For the CDs-UPy film with *α* of 0.89 mmol g^−1^, the recovery ratio of the CDs-UPy films from the second cycle to the fifth cycle decreased from 48.84 ± 2.66% to 34.40 ± 2.50%. At the same cycle, the recovery ratio of the CDs-UPy films decreased as *α* increased from 0.89 to 1.80 mmol g^−1^.

To study the effect of molecular weights of polyvinyl alcohol on the mechanical properties of CDs-UPy films, we prepared macroscopic films assembled from CDs-UPy with molecular weights of polyvinyl alcohol increasing from 9,000-10,000 to 130,000 g mol^−1^ (Supplementary Fig. 23a). The amounts of UPy on these CDs-UPy were ≈1.51 mmol g^−1^, which was also determined by the results of elemental analysis (Supplementary Table 1). The successful synthesis of the CDs-UPy made from polyvinyl alcohol of different molecular weights was confirmed with FTIR and ^1^H NMR (Supplementary Fig. 23b-f). The CDs-UPy made from polyvinyl alcohol of different molecular weights showed a dot-like shape with an average diameter of ≈4.0 nm (Supplementary Fig. 24). We investigated the mechanical properties of these CDs-UPy macroscopic films. Higher molecular weights led to the larger values of breaking strength, elongation at break, and *E* of the CDs-UPy macroscopic films. The *E* value increased from 299.77 ± 32.83 to 476.92 ± 86.82 MPa as molecular weight of polyvinyl alcohol increased from 9,000-10,000 to 130,000 g mol^−1^ (Fig. 3f). The breaking strength increased from 19.35 ± 0.97 to 31.49 ± 2.42 MPa with the molecular weight of polyvinyl alcohol increasing from 9,000-10,000 to 130,000 (Supplementary Fig. 25a). Moreover, the elongation at break increased from 67.52 ± 4.31% to 115.86 ± 7.79% as the molecular weight of polyvinyl alcohol increased from 9,000-10,000 to 130,000 (Supplementary Fig. 25b).

The CDs-UPy films with red or green fluorescence exhibited similar mechanical properties in comparison with CDs-UPy films (Supplementary Fig. 26a). For instance, the elongation at break of the CDs-1.80UPy films doped with red- or green-emissive CDs exhibited little variation, with the value of ≈50% (Supplementary Fig. 26b). Their breaking strength also exhibited little variation, with the value of ≈20 MPa (Supplementary Fig. 26c). Their *E* value was ≈400 MPa (Supplementary Fig. 26d). The good mechanical properties of the CDs-UPy macroscopic films can be further demonstrated through comparing mechanical properties of the CDs-UPy films with those of macroscopic materials assembled from other nanoparticles (Supplementary Table 3).

### Molecular dynamics simulation of CDs-UPy films

**Fig. 4:**
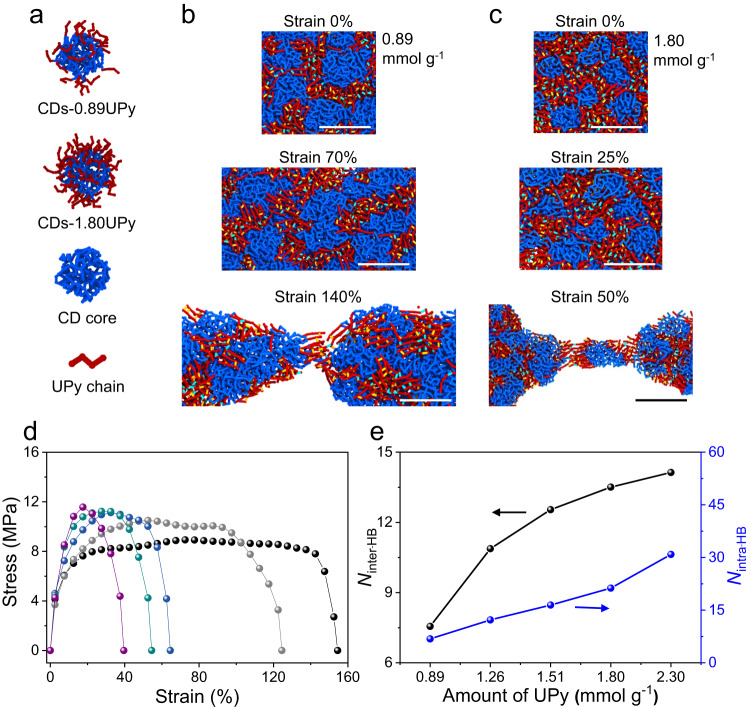
Molecular dynamics simulation of the CDs-UPy films. **a** Structures of CDs-UPy used in molecular dynamics simulation. **b**, **c** Structure evolution of CDs-UPy films with *α* of 0.89 (**b**) and 1.80 mmol g^−1^ (**c**) during the elongation process. The scale bar is 5 nm in **b**, **c**. The interparticle hydrogen bonding was labeled as light blue and the intraparticle hydrogen bonding was labeled as yellow. **d** The simulated stress−strain curves of CDs-UPy films with *α* of 0.89 (black), 1.26 (gray), 1.51 (dark blue), 1.80 (dark cyan), and 2.30 (purple) mmol g^-1^. **e** The numbers of the interparticle hydrogen bonding (*N*_inter-HB_) and the numbers of the intraparticle hydrogen bonding (*N*_intra-HB_) in CDs-UPy films with different *α* values. Source data are provided as a Source Data file.

To further understand the formation and mechanical properties of the CDs-UPy macroscopic films, molecular dynamics simulations were performed. The structure of CDs-UPy used in the molecular dynamics simulations was simplified as a carbon core (blue) grafted with certain number of UPy chains (red) (Fig. 4a and Supplementary Fig. 27). There existed the interparticle hydrogen bonding (light blue) and the intraparticle hydrogen bonding (yellow) to govern the formation of CDs-UPy films (Fig. 4b,c). The interparticle hydrogen bonding governed the formation of the CDs-UPy films. The intraparticle hydrogen bonding could cause the cross-linking in the CDs-UPy. The more intraparticle hydrogen bonding led to a higher degree of cross-linking in the CDs-UPy, making the CDs-UPy more rigid. After the formation of CDs-UPy films (strain=0%), the CDs-UPy deformed. The CDs-UPy with a higher *α* value showed a smaller deformation, in comparison with the CDs-UPy with a lower *α* value. The CDs-UPy films with higher *α* values exhibited a clearer boundary of CDs-UPy (Fig. 4b,c). With the further increase of the strain, the CDs-UPy films were elongated with the deformation of the CDs-UPy. More obvious deformation of CDs-UPy was observed in the CDs-UPy films with a lower *α* value (Fig. 4b,c). At the strain of breaking CDs-UPy films, the fracture of the interparticle hydrogen bonding was observed (Fig. 4b,c and Supplementary Movies 1, 2).

The interplay of the interparticle hydrogen bonding and the intraparticle hydrogen bonding played a crucial role in the mechanical properties of the CDs-UPy films. Figure 4d shows the simulated stress-strain curves of CDs-UPy films with different *α* values, which are qualitatively in agreement with the experimental results (Fig. 3a). With the increment of *α* values, the numbers of the interparticle hydrogen bonding and the intraparticle hydrogen bonding both increased (Fig. 4e). The increment of intraparticle hydrogen bonding led to an increase of cross-linking in CDs-UPy and thus the increase of the rigidity of the single CDs-UPy. Moreover, with the *α* value increasing, the numbers of interparticle hydrogen bonding increased from ≈8 to ≈14, resulting in a high density of cross-linking in CDs-UPy films and thus a large *E*. The decreased value of the elongation at break was caused by the increment of the cross-linking degree stemmed from the interparticle hydrogen bonding in CDs-UPy films. As *α* increased from 0.89 to 2.30 mmol g^−1^, the numbers of intraparticle hydrogen bonding increased from ≈7 to ≈31, leading to the increased rigidity of the CDs-UPy and thus the decreased elongation at break.

The molecular dynamics simulations were also helpful to understand the increase in *T*_g_ of the CDs-UPy films with *α* values increasing. As *α* increased from 0.89 to 2.30 mmol g^−1^, the number of interparticle hydrogen bonding and intraparticle hydrogen bonding increased, which led to the higher density of cross-linking in both CDs-UPy particles and macroscopic films. The higher density of cross-linking resulted in less movement of segments, thus leading to a higher *T*_g_ (Supplementary Fig. 19)^60–62^.

### Re-castability and self-healing properties of CDs-UPy films

**Fig. 5:**
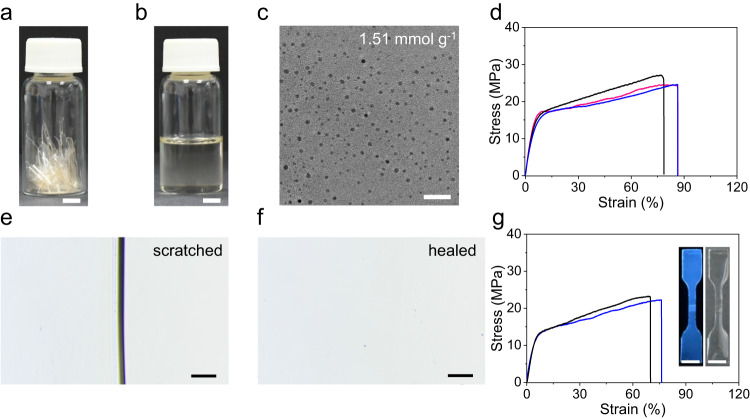
The re-castability and self-healing properties of the CDs-UPy films. **a** The photograph of fragments of the CDs-1.51UPy films. **b** The photograph of CDs−1.51UPy fragments dissolved in DMSO. The scale bars are 0.5 cm. **c** Representative TEM image of the CDs−1.51UPy after two-times recasting usages. The scale bar is 25 nm. **d** Stress−strain curves of CDs-UPy films with *α* of 1.51 mmol g^−1^ before (pink line) and after the first (black line) and second (blue line) recasting usage. **e** The photograph of scratch on the CDs-1.51UPy films. **f** The photograph of the healed scratch on the CDs-1.51UPy films. The scale bars are 200 μm. **g** Stress−strain curves of CDs-UPy films with *α* of 1.51 mmol g^−1^ before (blue line) and after (black line) welding. The inset shows the samples after being welded. The scale bars are 0.5 cm. Source data are provided as a Source Data file.

The CDs-UPy films with different *α* values were recast and reprocessed by dissolving the CDs-UPy films in the DMSO solvent (Fig. 5a,b). Figure 5c shows the representative TEM image of the CDs-1.51UPy after two-times recasting usages. The CDs-1.51UPy maintained a dot-like shape with an average diameter of ≈4.0 nm, indicating that the CDs-UPy showed no change after two-times recasting usages. Moreover, the CDs-UPy films with *α* varying from 0.89 to 2.30 mmol g^−1^ almost maintained their original mechanical properties after two-times recasting usages (Fig. 5d and Supplementary Fig. 28).

Furthermore, we studied the self-healing^63,64^ properties of the CDs-UPy films by both scratch healing and welding experiments. Scratches on the CDs-UPy films with *α* varying from 0.89 to 2.30 mmol g^−1^ were healed within 5 min upon heating in water at 60 ^o^C (Fig. 5e,f and Supplementary Fig. 29). We also performed welding experiments on the CDs-UPy films with *α* varying from 0.89 to 2.30 mmol g^−1^. The fully-cut samples were welded after heating at 60 ^o^C for 1 h under the pressure of 200 g weight and the assistance of the trace amount of water. The welded samples showed similar mechanical properties with original samples, indicating the excellent self-healing ability of the CDs-UPy films (Fig. 5g and Supplementary Fig. 30). Moreover, through welding experiments, we showed a proof-of-concept actuating application of CDs-UPy films triggered by the humidity (Supplementary Fig. 31 and Supplementary Note 2).

### Shape-programming of CDs-UPy films by hydrosetting

**Fig. 6:**
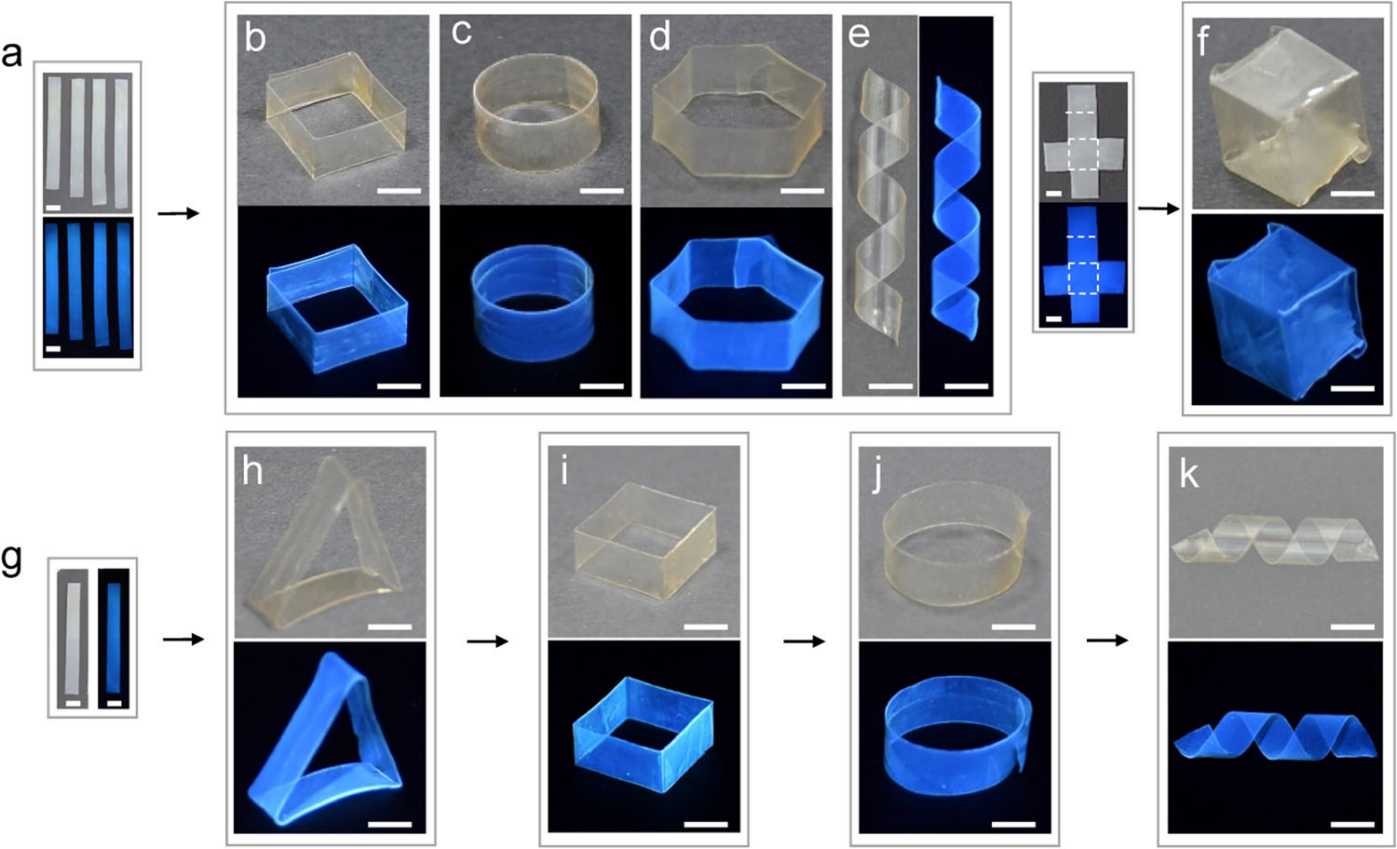
Shape-programmable hydrosetting of CDs-UPy films. **a** Photographs of stripes of CDs-UPy films immersed in the water. **b**–**e** Photographs of diverse programmed shapes of CDs-UPy film stripes. **f** The photograph of the cube (right) folded from the cross-shaped CDs-UPy films along dashed lines (left). **h**–**k** The sequential shaping of the same CDs-UPy film stripe (**g**) into a triangle (**h**), a square (**i**), a ring (**j**), and a helix (**k**). Scale bars are 5 mm. *α* = 1.51 mmol g^−1^. The photographs were taken under the ambient light (up in **a**–**d**, **f**, **h**–**k**, and left in **e**, **g**) and the ultraviolet lamp (down in **a**–**d**, **f**, **h**–**k**, or right in **e**, **g**).

Upon swelling in the water, the CDs-UPy films absorbed the water (Fig. 2k), which softened the CDs-UPy films. The *E* of the CDs-1.51UPy film in the water decreased to 0.08 folds of its *E* value of the dry film. The *E* of the CDs-UPy films in the water increased from 2.52 ± 1.04 to 229.68 ± 68.34 MPa as the *α* value increased from 0.89 to 2.30 mmol g^−1^ (Supplementary Fig. 32). The CDs-UPy films were simply processed into diverse shapes via a sustainable, environmental-friendly, and facile hydrosetting method^65–67^. First, the CDs-UPy stripes were immersed in the water for ≈10 min to soften the CDs-UPy films (Fig. 6a). Subsequently, the CDs-UPy stripes in the wet state were processed into programmed shapes by the aid of different molds (Supplementary Fig. 33). After drying at the ambient environment (room temperature and ≈30% humidity), fixed shapes of CDs-UPy films were obtained by releasing them from the molds, including square, circle, hexagon, and helix (Fig. 6b-e). Moreover, a cube of the CDs-UPy film was constructed by folding the cross-shaped CDs-UPy films via the hydrosetting method (Fig. 6f).

Additionally, a stripe of the CDs-UPy film was sequentially processed into various shapes via the hydrosetting method. For instance, a stripe of the CDs-UPy film (Fig. 6g) was transformed into a triangle, a square, a ring, and a helix in programmable sequences via the hydrosetting process (Fig. 6h-k). Notably, due to the inheritance of fluorescence properties from the pristine CDs, the programmed shapes of CDs-UPy films also showed blue fluorescence under the ultraviolet lamp. Furthermore, the CDs-UPy films with different fluorescent colors were also processed into programmed shapes, such as helices with red or green emission (Supplementary Fig. 34). These shapes of CDs-UPy films were stable for at least 1 year.

## Discussion

In summary, we report a recastable, self-healing, hydrosetting, and mechanically robust macroscopic material assembled from the CDs-UPy solely driven by hydrogen bonds. The CDs-UPy films showed fluorescent properties that were inherited from the original CDs. The fluorescent colors of the macroscopic films were tuned through the combination of the CDs-UPy with other CDs. The CDs-UPy films exhibited unexpected collective properties, such as re-castability, self-healing, hydrosetting ability, and mechanical robustness, in comparison with the macroscopic materials assembled from other nanoparticles^1^. Through the precise and quantitative UPy modification on the nanoscale CDs to master the interaction forces between CDs-UPy, the macroscopic CDs-UPy films exhibited tunable mechanical properties. Furthermore, molecular dynamics simulations unveiled that the interplay of the interparticle hydrogen bonding and the intraparticle hydrogen bonding governed the formation and mechanical properties of the CDs-UPy macroscopic films.

Notably, through a hydrosetting method, the CDs-UPy films were processed into programmable shapes by using water to plasticize the films without using the harsh processing conditions and expensive and complicated processing machines. The results in this work uncover an alternative class of mechanically robust macroscopic materials with the environmental-friendly CDs as building blocks, making its processing and recasting in a sustainable and eco-friendly fashion. Because of these good properties, CDs-UPy films may have the potential applications in flexible sensors, soft robots, and optical coatings^1,68^. The methodology in this work can be generally applied to other types of macroscopic materials assembled/coassembled from various CDs and functional nanoparticles to achieve functional macroscopic materials and devices with improved mechanical performances, re-castability, and sustainable processability.

## Methods

### Materials

Polyvinyl alcohol (PVA, *M*_w_, 13,000 g mol^−1^, 98% hydrolyzed) was purchased from Acros Organics. PVA (*M*_w_, 9000–10,000 g mol^−1^, 80% hydrolyzed, *M*_w_, 31,000–50,000 g mol^−1^, 98–99% hydrolyzed, *M*_w_, 89,000–98,000 g mol^−1^, 99% hydrolyzed, *M*_w_, 130,000 g mol^−1^, 99% hydrolyzed) was purchased from Sigma-Aldrich. 2-Amino-4-hydroxy-6-methylpyrimidine (98%) was purchased from Sigma-Aldrich. 1,6-Diisocyanatohexane (>98%) was purchased from TCI. Dibutyltin dilaurate (98%) was purchased from Energy Chemical. Anhydrous dimethyl sulfoxide (DMSO, 99.9%) was purchased from J&K Chemical. Chloroform (>99%), N, N-dimethylformamide (DMF, > 99%), and ethanol (>99%) were purchased from Beijing Chemical Reagents. Citric acid (>99.5%), urea (>99.5%), and sodium hydrate (NaOH, 99.9%) were purchased from Aladdin. All the chemicals were used without further purification. Deionized (DI) water with a resistance of 18.2 MΩ cm was used in all experiments.

### Preparation of CDs

6.16 g PVA was added to the 200 mL DI water. The suspension was stirred and heated at 100 ^o^C until the complete dissolution of the PVA. The PVA solution (65 mL) was transferred to a poly(tetrafluoroethylene) (Teflon)-lined stainless-steel autoclave (100 mL) and heated at 200 °C for 6 h in an oven. After cooling the autoclave to room temperature, the light yellow solution was filtered through a 0.8 μm microporous membrane. The filtered solution was dialyzed against DI water (4000 mL) through a dialysis membrane (*M*_w_ cut-off 50 kg mol^−1^) for 7 days and the DI water was changed three times every day. The purified solution was concentrated by a rotary evaporator to remove excess water. After freeze-drying, the solid of the CDs was obtained. Yield: 69.79%. ^13^C NMR (125 MHz, DMSO-*d*_6_) δ 68.05, 67.97, 67.77, 67.56, 66.08, 66.01, 65.92, 65.85, 65.78, 65.70, 64.03, 63.85, 63.69, 46.20, 45.80, 45.31, 44.68.

The synthesis of the red- or green-emissive CDs was carried out according to a reported method^69^. Citric acid (1 g) and urea (2 g) were added to a Teflon-lined stainless-steel autoclave containing 10 mL DMF and then the solution reacted at 160 °C for 4 h under the solvothermal condition. The resulting solution was mixed with 20 mL NaOH (1 M) aqueous solution and then centrifuged at 10,190× *g* for 15 min. The precipitates were washed with DI water twice and collected for lyophilization to obtain the solid of red-emissive CDs.

Citric acid (1 g) and urea (2 g) were added to 10 mL ethanol and then the solution reacted at 160 °C for 4 h under the solvothermal condition. The resulting solution was mixed with 20 mL ethanol and then centrifuged at 10,190× *g* for 15 min. The precipitates were washed with ethanol twice and collected for drying under vacuum at 60 °C to obtain the solid of green-emissive CDs.

### Synthesis of UPy-NCO

The synthesis of UPy-NCO was carried out according to a reported method^70^. 2-amino-4-hydroxy-6-methylpyrimidine (2.5 g, 20 mmol) and 1,6-diisocyanatohexane (22.425 mL, 140 mmol) were mixed in a 100 mL round-bottom flask with a reflux condenser. The mixture was stirred and heated at 100 °C for 12 h under the nitrogen atmosphere. After cooling, pentane (60 mL) was added to the mixture. The precipitates were collected by the vacuum filtration and washed with pentane (60 mL) for 3 times. Finally, the precipitates were dried in a vacuum oven overnight to obtain UPy-NCO. Yield: 93.91%. ^1^H NMR (500 MHz, CDCl_3_) δ 13.10 (s, 1H, CH_3_CN*H*), 11.85 (s, 1H, CH_2_NH(C = O)N*H*), 10.17 (s, 1H, CH_2_N*H*(C = O)NH), 5.81 (s, 1H, C*H* = CCH_3_), 3.33 – 3.21 (m, 4H, NH(C = O)NHC*H*_*2*_ + C*H*_*2*_NCO), 2.23 (s, 3H, C*H*_*3*_C = CH), 1.65–1.57 (m, 4H, NCH_2_C*H*_*2*_), 1.45 – 1.34 (m, 4H, CH_2_CH_2_C*H*_*2*_CH_2_CH_2_). ^13^C NMR (125 MHz, DMSO-*d*_*6*_) δ 164.79, 161.12, 154.78, 151.41, 121.51, 104.52, 42.49, 38.91, 30.45, 28.99, 25.61, 25.60, 23.24.

### Preparation of the CDs-UPy

The detailed mass formula of the CDs and UPy-NCO for the preparation of CDs-UPy with the different modification amounts of UPy groups, *α*, was shown in Supplementary Table 2. For example, 2.2 g CDs, 2.0 g UPy-NCO, and 0.4 mL dibutyltin dilaurate were added into a round-bottom flask containing 200 mL anhydrous DMSO. The mixed solution was stirred and heated at 80 °C for 6 h under the nitrogen atmosphere. After cooling to the room temperature, the reaction solution was added into 2 L chloroform and the precipitates were collected by the vacuum filtration. The precipitates were repeatedly washed with chloroform and DI water. Subsequently, the precipitates were dialyzed in 2 L DI water for 24 h. The solid of the CDs-1.51UPy was obtained by the lyophilization.

### Preparation of the macroscopic CDs-UPy films

250 mg CDs-UPy was added into 5 mL DMSO and the suspension was stirred and heated at 80 °C until the complete dissolution of CDs-UPy. A 5 mL CDs-UPy solution (50 mg mL^−1^) was added into a glass petri dish with a diameter of ≈6 cm and DMSO was removed at 60 °C for 24 h. The CDs-UPy films with a diameter of ≈6 cm were peeled off from the glass substrate and the residual solvent in the films was removed under the vacuum condition at 60 °C for 24 h.

To prepare the red-emissive CDs doped CDs-UPy films, 1 mg red-emissive CDs was added to a 5 mL DMSO solution containing 50 mg mL^−1^ CDs-1.80UPy. After stirring at 80 °C for 10 min, the 5 mL mixed solution of CDs-1.80UPy and red-emissive CDs was added into a glass petri dish with a diameter of ≈6 cm and DMSO was removed at 60 °C for 24 h. The red-emissive CDs doped CDs-UPy films with a diameter of ≈6 cm were peeled off from the glass substrate and the residual solvent in the films was removed under the vacuum condition at 60 °C for 24 h.

To prepare the green-emissive CDs doped CDs-UPy films, 8 mg green-emissive CDs was added to a 5 mL DMSO solution containing 50 mg mL^−1^ CDs-1.80UPy. After stirring at 80 °C for 10 min, a 5 mL mixed solution of CDs-1.80UPy and green-emissive CDs was added into a glass petri dish with a diameter of ≈6 cm and DMSO was removed at 60 °C for 24 h. The green-emissive CDs doped CDs-UPy films with a diameter of ≈6 cm were peeled off from the glass substrate and the residual solvent in the films was removed under the vacuum condition at 60 °C for 24 h.

### Characterization of the CDs, UPy-NCO, and CDs-UPy

Elemental analysis was performed on Elementar Vario MICRO CUBE. NMR spectra were recorded on a Bruker AVANCE III (500 MHz, ^1^H; 125 MHz, ^13^C) NMR spectrometers at room temperature. Chemical shifts for ^1^H spectra were referenced to the residual solvent peak (CDCl_3_: ^1^H, 7.26). Chemical shifts for ^13^C spectra were referenced to the solvent peak (DMSO-*d*_6_: ^13^C, 39.52). The *T*_2_ was determined by Carr-Purcell-Meiboom-Gill (CPMG) NMR measurements. The sweep width and O1 were set as 10 and 3 ppm, respectively. The recovery time was set as 5, 10, 20, 30, 50, 100, 200, 350, 500, 1000, 2000, and 5000 ms. The protons of PVA (4 to 5.2 ppm) and UPy motifs (5.76 ppm) were chosen for analysis by integrating the peak area in the software of Origin. The area of the NMR peak of PVA motifs from the CDs and CDs-UPy at each recovery time was fitted with a double-exponential decay to obtain the *T*_2_. The area of the NMR peak of UPy motifs from the CDs-UPy at each recovery time was fitted with a mono-exponential decay to obtain the *T*_2_.

Infrared spectra of the CDs, UPy-NCO, and CDs-UPy were recorded using an attenuated total reflectance Fourier transform infrared spectroscopy (ATR-FTIR, Thermo Fisher Scientific Nicolet iS10, Thermo Fisher Scientific Smart iTR* ATR with a diamond crystal). All FTIR spectra were recorded between 4000 and 400 cm^−1^ with a resolution of 4 cm^−1^ and 32 scans. The morphology of the CDs and CDs-UPy was measured by using TEM. 2 μL of the CDs (0.5 mg mL^−1^) or CDs-UPy solution (0.5 mg mL^−1^) was drop-cast on an ultrathin carbon film on the TEM grid at room temperature. JEM-2100F electron microscope was used to obtain TEM images at 200 kV with a CCD camera. The obtained images were analyzed by using a software ImageJ.

Moreover, the AFM image of the CDs-UPy was taken in the tapping mode by the Bruker’s Dimension FastScan AFM under ambient condition. To prepare the sample, 10 μL of the CDs-UPy solution (0.04 mg mL^−1^) was spin-coated on a freshly cleaved mica substrate at 3000 rpm for 1 min. The AFM images were analyzed by using the NanoScope Analysis software of Bruker. The CDs-UPy films were adhered to the silicon wafer using double sided adhesive tape for AFM measurements. The roughness values were directly calculated by the NanoScope Analysis software of Bruker by using the equations provided in user manual. *R*a, arithmetic average of the absolute values of the surface height deviations is calculated from ${R}_{{{{{{\rm{a}}}}}}}=\frac{1}{N}\mathop{\sum }\nolimits_{j=1}^{N}|{Z}_{j}|$. *R*q, root mean square average of height deviations is calculated from ${R}_{{{{{{\rm{q}}}}}}}=\sqrt{\frac{\sum {{Z}_{i}}^{2}}{N}}$.

The *T*_g_ was determined on a TA Instruments Q20 differential scanning calorimetry under the nitrogen flow of 50 mL min^−1^. Solid samples (≈ 5 mg) were first heated to 180 °C at a rate of 10 °C min^−1^ and held at 180 °C for 2 min. Subsequently, the samples were cooled down to −20 °C at a rate of 10 °C min^−1^ and equilibrated at this temperature for 2 min. The DSC curves were obtained by reheating samples from −20 °C to 180 °C at a rate of 10 °C min^−1^. PL spectra were performed on a RF-5301 PC spectrophotometer (Shimadzu). UV-vis absorption spectra were obtained using a Shimadzu 3100 UV-vis spectrophotometer. Matrix-assisted laser desorption/ionization time-of-flight mass spectroscopy (MALDI-TOF MS) was performed on a Autoflex speed TOF/TOF mass spectrometer (Bruker Daltonics, Germany) with a pulsed Nd:YAG laser (355 nm). The raw data was processed in the FlexAnalysis software. The hydrodynamic diameters of the CDs were measured on a Malvern Zetasizer pro at 25 °C. Size by number was calculated by the software Malvern ZS XPLORER. Raman spectra were recorded using a Horiba LabRAM HR Evolution spectrometer with a 532 nm laser.

### Characterization of the macroscopic CDs-UPy films

Photographs of the CDs-UPy macroscopic films under the ambient light and the ultraviolet lamp (365 nm) were taken by a digital camera (Nikon D7500). The morphology of the CDs-UPy macroscopic films was investigated by scanning electron microscopy. The CDs-UPy films were freeze-fractured in the liquid nitrogen to obtain freshly fractured surfaces. The CDs-UPy films were sputtered with a thin layer of platinum prior to the SEM imaging. SU-8020 scanning electron microscope was used to obtain SEM images of the CDs-UPy films at an acceleration voltage of 5 kV. Swelling and dissolution of the CDs-UPy films were performed by immersing samples into 10 mL solvents separately, including ethyl acetate (EA), tetrahydrofuran (THF), diethyl ether (DEE), n-hexane (n-Hex), dichloromethane (DCM), acetone (DMK), ethanol (EtOH), toluene (TOL), acetonitrile (ACN), trichloromethane (TCM), and DI water at 25 °C for 24 h. The samples were taken out to weigh (*m*_swollen_) after gently removing the excess solvents on the surface of the films with the filter paper. Subsequently, the samples were dried under vacuum at 60 °C for 24 h and weighed as *m*_dry_. The swelling ratio and weight maintenance of the CDs-UPy films in different solvents were calculated based on the following equations:

1 $${{{{{\rm{Swelling}}}}}}\,{{{{{\rm{ratio}}}}}}=({m}_{{{{{{\rm{swollen}}}}}}}-{m}_{{{{{{\rm{dry}}}}}}})/{m}_{{{{{{\rm{dry}}}}}}}$$

2 $${{{{{\rm{Weight}}}}}}\,{{{{{\rm{maintenance}}}}}}={m}_{{{{{{\rm{dry}}}}}}}/{m}_{{{{{{\rm{initial}}}}}}}$$

### Mechanical properties of the CDs-UPy films

The uniaxial tensile tests of the CDs-UPy macroscopic films were conducted on the universal material testing machine (MTS Exceed Model E43) with a 20 N load cell at a loading rate of 10 mm min^−1^. Dumbbell shape specimens die-cut from the CDs-UPy macroscopic films with a width of 2 mm and a gauge length of 12 mm were used for tensile measurement. Cyclic tensile loading–unloading tests at a fixed strain of 20% were performed immediately after the first loading–unloading cycle on fresh specimens. All the tests were performed at room temperature with relative humidity at ≈30-40%. At least three individual specimens were tested. The tensile Young’s modulus was determined from the slope of the linear portion in the tensile stress-strain curves. Toughness was calculated as the integral area under the stress–strain curve. The energy dissipation of each cycle was calculated by integrating the area of hysteresis loop. The recovery ratio was calculated as the ratio of the dissipated energy in each cycle to that of the first cycle.

### Coarse-grained molecular dynamics simulations

We performed coarse-grained (CG) molecular dynamics simulations to understand the roles of the hydrogen bonding interactions in CDs-UPy films on their mechanical properties through uniaxial tension tests. Specifically, a CD is modeled as a cross-linked spherical network that consists of 250 CG beads that are assigned to type A (Supplementary Fig. 27). The network is constructed by packing a linear chain into a sphere and randomly cross-linking it to keep the spherical shape. Meanwhile, the network shows the deformability of CDs. We found the softness of CDs with ≈100 cross-linking bonds per CD can well agree with experimental measurement of mechanical properties of the CDs-UPy films. A UPy chain is represented by four linearly connected CG beads in the sequence of B-C-B-D (Supplementary Fig. 27). The interactions of CG beads in our model refer to Martini force field^71^. Hydrogen bonding interactions are explicitly described by the dynamic bonds that can form and break over time.

In coarse-grained (CG) simulations, the reduced units for length σ_0_, energy ϵ_0_, mass m_0_, and time *τ*_0_ are 1.0 nm, 1.0 kJ mol^−1^, 1.0 atomic mass unit, and 1.0 ps, respectively. Van der Waals interactions were defined as non-bonded interactions which are described by the Lennard-Jones potential,

3 $${V}_{{{\mbox{LJ}}}}(r)=4.0{\epsilon }_{{ij}}\left[{\left(\frac{{\sigma }_{{ij}}}{{r}_{{ij}}}\right)}^{12}-{\left(\frac{{\sigma }_{{ij}}}{{r}_{{ij}}}\right)}^{6}\right]$$

where *r*_*ij*_ is the distance between bead *i* and bead *j* and the interaction parameters *ϵ*_*ij*_ and *σ*_*ij*_ are listed in Supplementary Table 4. Harmonic potential is employed to describe interactions of bond stretching,

4 $${V}_{{{{{{\rm{bond}}}}}}}(r)=\frac{1}{2}{K}_{{{{{{\rm{bond}}}}}}}{(r-{r}_{0})}^{2}$$

where ${r}_{0}$ = 0.47 *σ*_0_ and ${K}_{{{{{\rm{bond}}}}}}$ = 1250 ϵ_0_/*σ*_0_^2^. The hydrogen bonding interactions also employ the form of harmonic potential. The formation and breaking of hydrogen bonds are simulated by Monte Carlo method^72^ with the probabilities of ${P}_{f}$ and ${P}_{b}$, respectively, which are associated to hydrogen bond strength ${V}_{{{{{{\rm{HB}}}}}}}$,

5 $${P}_{f}=\left\{\begin{array}{cc}\exp [-({V}_{{{{{{\rm{bond}}}}}}}(r)-{V}_{{{{{{\rm{HB}}}}}}}){/{{{{{\rm{k}}}}}}}_{{{{{{\rm{B}}}}}}}{{{{{\rm{T}}}}}}],& {V}_{{{{{{\rm{bond}}}}}}}(r) \, > \, {V}_{{{{{{\rm{HB}}}}}}}\\ 1.0,\hfill & {V}_{{{{{{\rm{bond}}}}}}}(r)\, \le \, {V}_{{{{{{\rm{HB}}}}}}}\end{array}\right.$$

6 $${P}_{b}=\left\{\begin{array}{cc}\exp [({V}_{{{{{{\rm{bond}}}}}}}(r)-{V}_{{{{{{\rm{HB}}}}}}}){/{{{{{\rm{k}}}}}}}_{{{{{{\rm{B}}}}}}}{{{{{\rm{T}}}}}}],& \,{V}_{{{{{{\rm{bond}}}}}}}(r) \, < \, {V}_{{{{{{\rm{HB}}}}}}}\\ 1.0,\hfill& \,{V}_{{{{{{\rm{bond}}}}}}}(r)\, \ge \, {V}_{{{{{{\rm{HB}}}}}}}\end{array}\right.$$

where ${V}_{{{{{{\rm{HB}}}}}}}$ is set as 20 kJ mol^−1^ according to the calculation of the DFT^73^. The hydrogen bonds present between the two CG beads with the types of B and B, or D and D, or B and D.

The interactions of angle bending of UPy groups are described by the harmonic potential,

7 $${V}_{{{{{{\rm{angle}}}}}}}(r)=\frac{1}{2}{K}_{{{{{{\rm{angle}}}}}}}{(\theta -{\theta }_{0})}^{2}$$

where *K*_angle_ = 25 *ϵ*_0_, *θ*_0_ = π for ∠ABC, ∠BCB, and ∠CBD, and *θ*_0_ = π/2 for ∠AAB.

The beads are integrated according to the Newton’s equations of motion with a time step d*t* = 0.01*τ*_0_. A nanoparticle is composed of a CD core and grafted UPy groups. We construct the pure CDs-UPy systems with 150, 125, 110, 100, and 90 nanoparticles each of which is grafted with 24, 38, 47, 56, and 72 UPy groups, respectively, to keep the approximate volume of the different CDs-UPy systems. The UPy chains are randomly grafted on the surface of CDs. The nanoparticles are initially randomly placed in a simulation box under three-dimensional periodic boundary conditions. The equilibrium structure is obtained by running the simulations of 20 ns under an isothermal-isobaric ensemble. In the simulation of tension tests, the periodic boundary conditions in *X* and *Y* directions are removed, while the periodic condition in *Z* direction is kept^74^. The uniaxial tension simulations are performed in *Z* direction by a constant tension rate of 2.5×10^−7^ strain unit per molecular dynamics step with the maximum strain of 200%. The stress is estimated by the pressure multiplied by the initial cross-sectional area of the sample that is consistent with experimental measurement. The CG simulations are all performed on GPU by an in-house MD package^75,76^.

### Recasting and self-healing of CDs-UPy films

The CDs-UPy films were recast by adding into 5 mL DMSO and the suspension was stirred and heated at 80 °C until the complete dissolution of films. The re-dissolved solution was used to prepare CDs-UPy films again. The above process was repeated at least twice. The scratches in the scratch healing tests were made by scratching on the surface of the CDs-UPy films with blunt head tweezers. The CDs-UPy films with scratches were heated in water at 60 ^o^C. After 5 min, the scratches on the CDs-UPy films were observed to be healed by optical microscopy. In the welding experiments, the fully-cut dumbbell shape samples were overlapped 2 mm in length and heated at 60 ^o^C under the pressure of 200 g weight and the assistance of the trace amount of water. After 1 h, the CDs-UPy films were welded as dumbbell shape samples with a width of 2 mm and a gauge length of 10 mm for tensile measurement.

### Shape-programming of CDs-UPy films by hydrosetting

The stripes with a width of ≈5 mm and a length of ≈4-5.5 cm as well as the cross-shaped films were cut from the CDs-UPy films. They were immersed in the DI water at room temperature for ≈10 min. The wet state stripes were processed into different shapes by the aid of molds. After drying at room temperature and ≈30% relative humidity for ≈2 h, fixed shapes of CDs-UPy films were obtained after the removal from the molds. To sequentially shape the same CDs-UPy film stripe into different shapes, a triangle of the CDs-UPy films was first constructed. Subsequently, the triangle was immersed in the DI water at room temperature for ≈10 min to recover its stripe shape. This stripe was shaped into a square of CDs-UPy film by using the same method. A ring and a helix of CDs-UPy film were then shaped by repeating the above experimental procedure for shaping.

The protocol of programming the CDs-UPy films with different fluorescence colors into helix shapes with red or green emission is identical to the above experimental procedure. Photographs of the stripes, cross-shaped films, and fixed shapes of CDs-UPy films under the ambient light and the ultraviolet lamp (365 nm) were taken by a digital camera.

### Supplementary information

Supplementary Information

Description of Additional Supplementary Files

Supplementary Movie 1

Supplementary Movie 2

### Source data

Source Data

## Data Availability

The data that support the findings of this study are available from the corresponding author upon request. Source data are provided with this paper.

## Code availability

The code that support the findings of this study are available from the corresponding author upon request.The package for the MD simulations is available via GitHub at https://github.com/youliangzhu/pygamd-v1 and Zenodo^77^. The input files for MD simulations and the codes for data analysis are available via GitHub at https://github.com/youliangzhu/carbon-dots.git and Zenodo^78^.
