# Supplementary material for: Recastable assemblies of carbon dots into mechanically robust macroscopic materials
Source: Nat Commun. 2023 Oct 25;14:6782. doi: 10.1038/s41467-023-42516-8 (PMC10600192; doi:10.1038/s41467-023-42516-8)
Supplement: Supplementary file 1 — Supplementary Information [file 41467_2023_42516_MOESM1_ESM.pdf]

Supplementary Information

Recastable assemblies of carbon dots into mechanically robust macroscopic materials

Bowen Sui[1,#], Youliang Zhu[1,#], Xuemei Jiang[1], Yifan Wang[1], Niboqia Zhang[1], Zhongyuan Lu[1], Bai Yang[1], Yunfeng Li[1,*]

[1]State Key Laboratory of Supramolecular Structure and Materials, College of Chemistry, Jilin University, Changchun, 130012, China.

[#]These authors contributed equally: Bowen Sui, Youliang Zhu

[*]E-mail: yflichem@jlu.edu.cn

Supplementary Figures

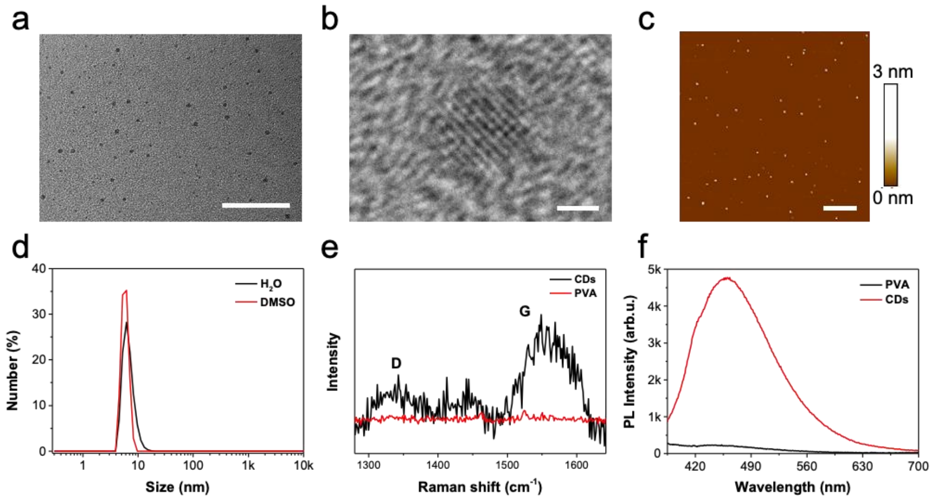

Supplementary Figure 1. Characterization of the pristine CDs. (a) TEM and (b) high-resolution TEM (HR-TEM) images of the pristine CDs. The scale bar in (a) is 50 nm. The scale bar in (b) is 1 nm. (c) Representative AFM images of the pristine CDs. The scale bar is 500 nm. (d) The size distribution of the CDs in H_2O and DMSO solution by DLS measurement. (e) Raman spectrum of the CDs and PVA. (f) The photoluminescence (PL) spectra of the CDs and PVA in water with the same concentration of 5 mg mL^{-1}. Source data are provided as a Source Data file.

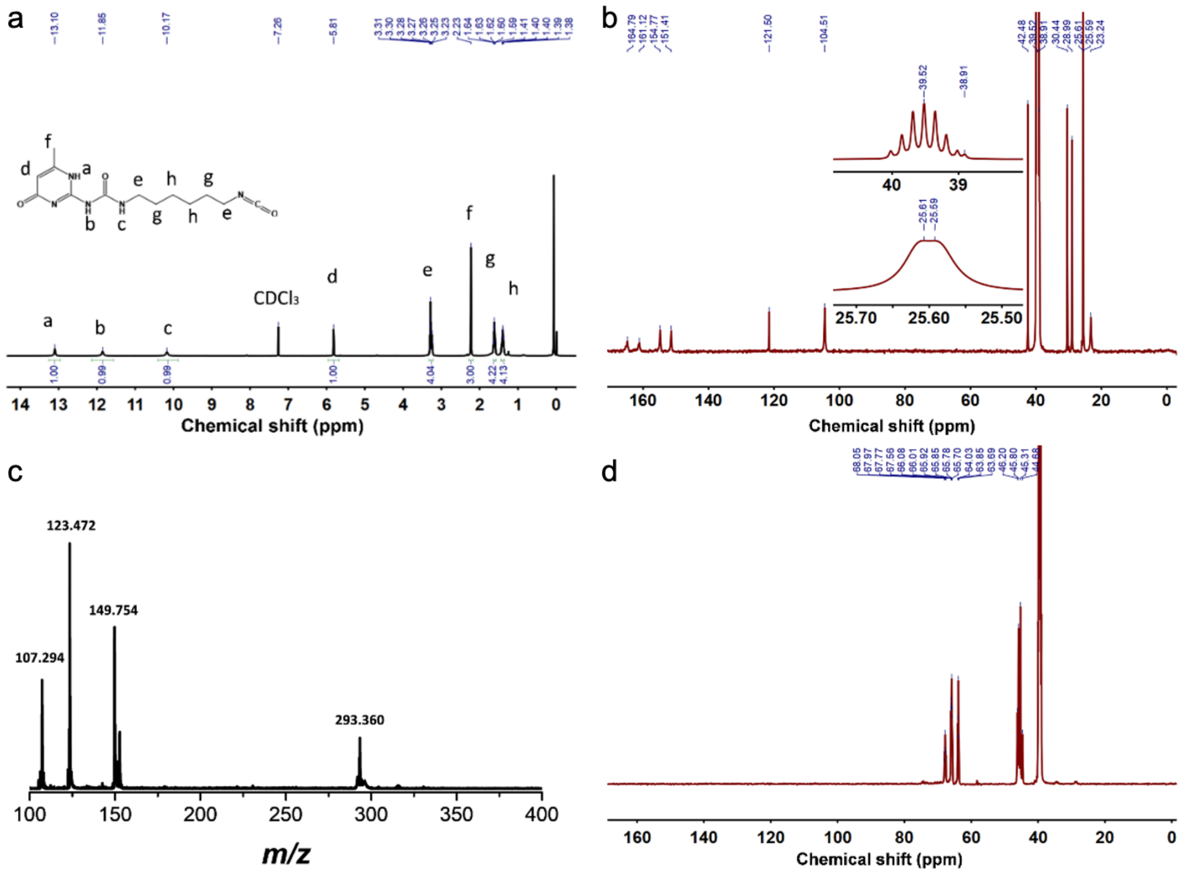

Supplementary Figure 2. Characterization of UPy-NCO and the pristine CDs. (a) ^{1}H NMR spectrum of UPy-NCO with $CDCl_3$ as solvent. (b) ^{13}C NMR spectrum of UPy-NCO with DMSO-d_6 as solvent. (c) MALDI-TOF MS spectrum of the UPy-NCO. (d) ^{13}C NMR spectrum of the pristine CDs with DMSO-d_6 as solvent. Source data are provided as a Source Data file.

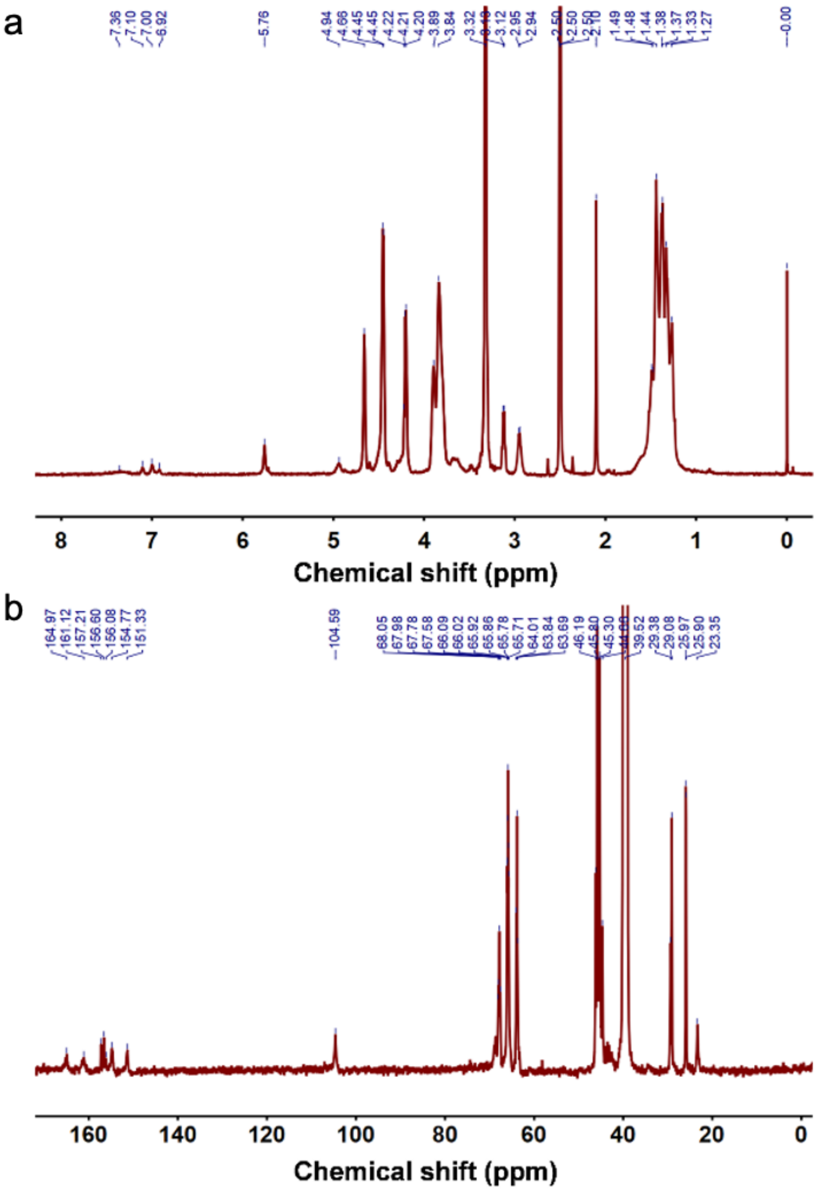

Supplementary Figure 3. NMR characterization of CDs-0.89UPy. (a) ^{1}H and (b) ^{13}C NMR spectrum of CDs-0.89UPy with DMSO-d_6 as solvent. Source data are provided as a Source Data file.

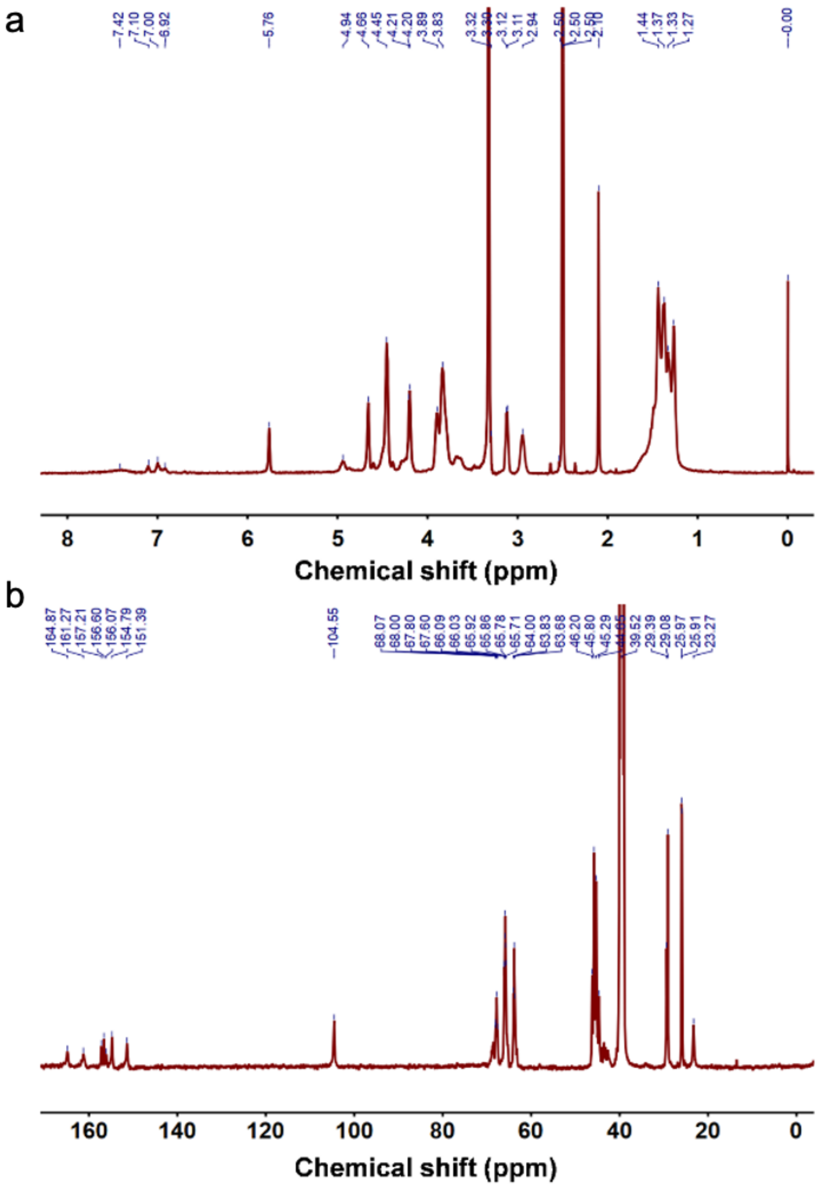

Supplementary Figure 4. NMR characterization of CDs-1.26UPy. (a) ^{1}H and (b) ^{13}C NMR spectrum of CDs-1.26UPy with DMSO-d_6 as solvent. Source data are provided as a Source Data file.

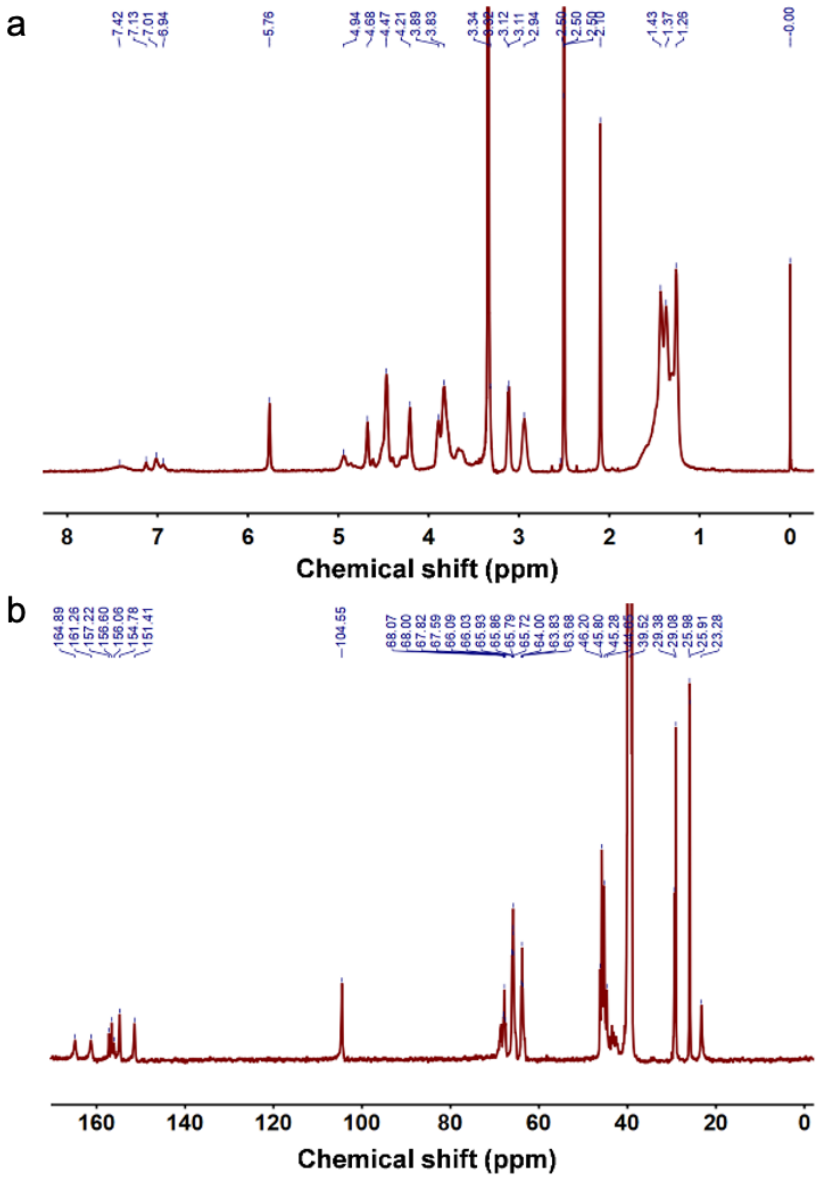

Supplementary Figure 5. NMR characterization of CDs-1.51UPy. (a) ^{1}H and (b) ^{13}C NMR spectrum of CDs-1.51UPy with DMSO-d_6 as solvent. Source data are provided as a Source Data file.

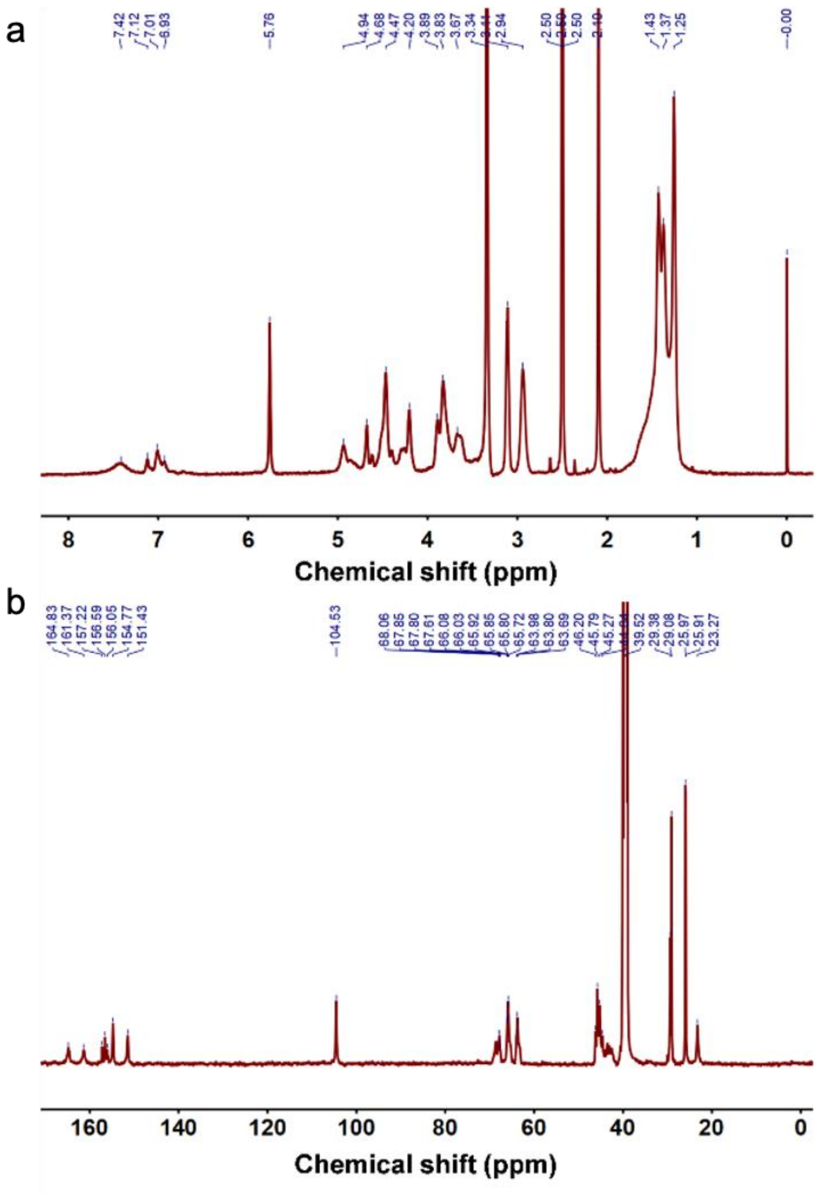

Supplementary Figure 6. NMR characterization of CDs-1.80UPy. (a) ^{1}H and (b) ^{13}C NMR spectrum of CDs-1.80UPy with DMSO-d_6 as solvent. Source data are provided as a Source Data file.

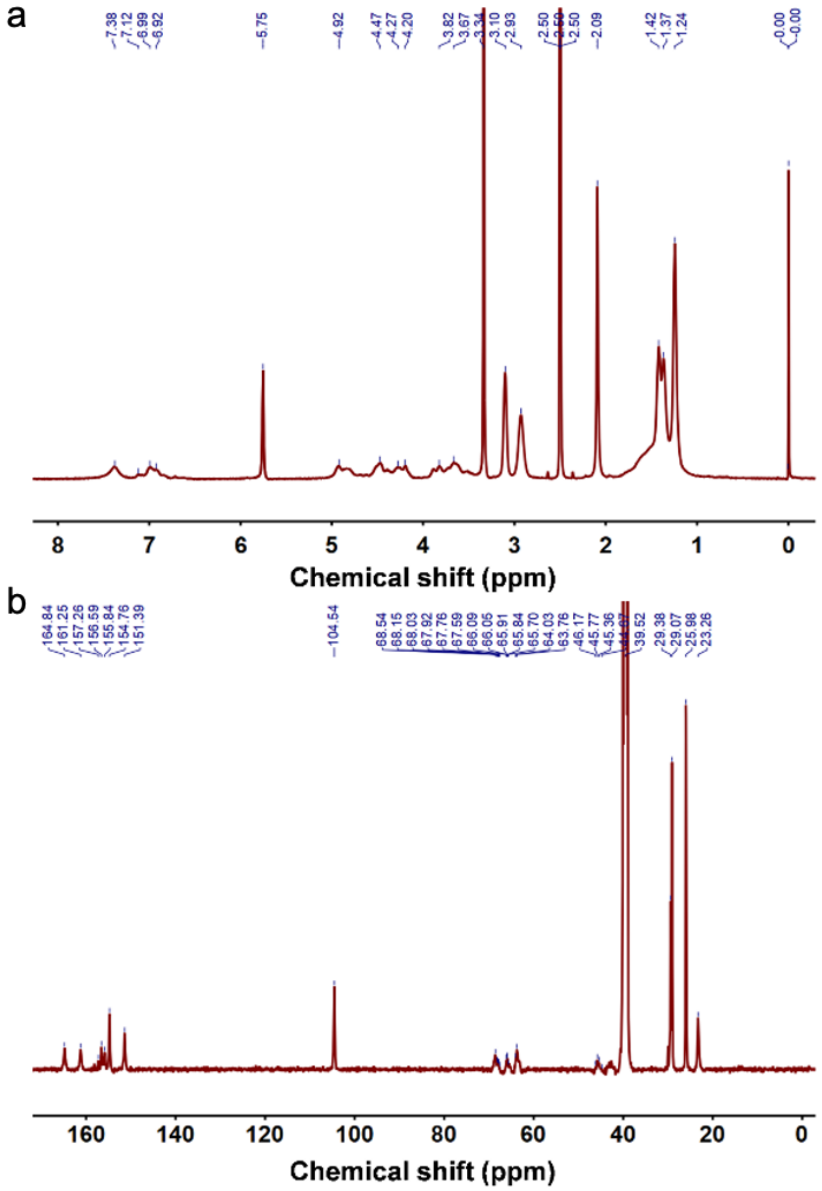

Supplementary Figure 7. NMR characterization of CDs-2.30UPy. (a) ^{1}H and (b) ^{13}C NMR spectrum of CDs-2.30UPy with DMSO-d_6 as solvent. Source data are provided as a Source Data file.

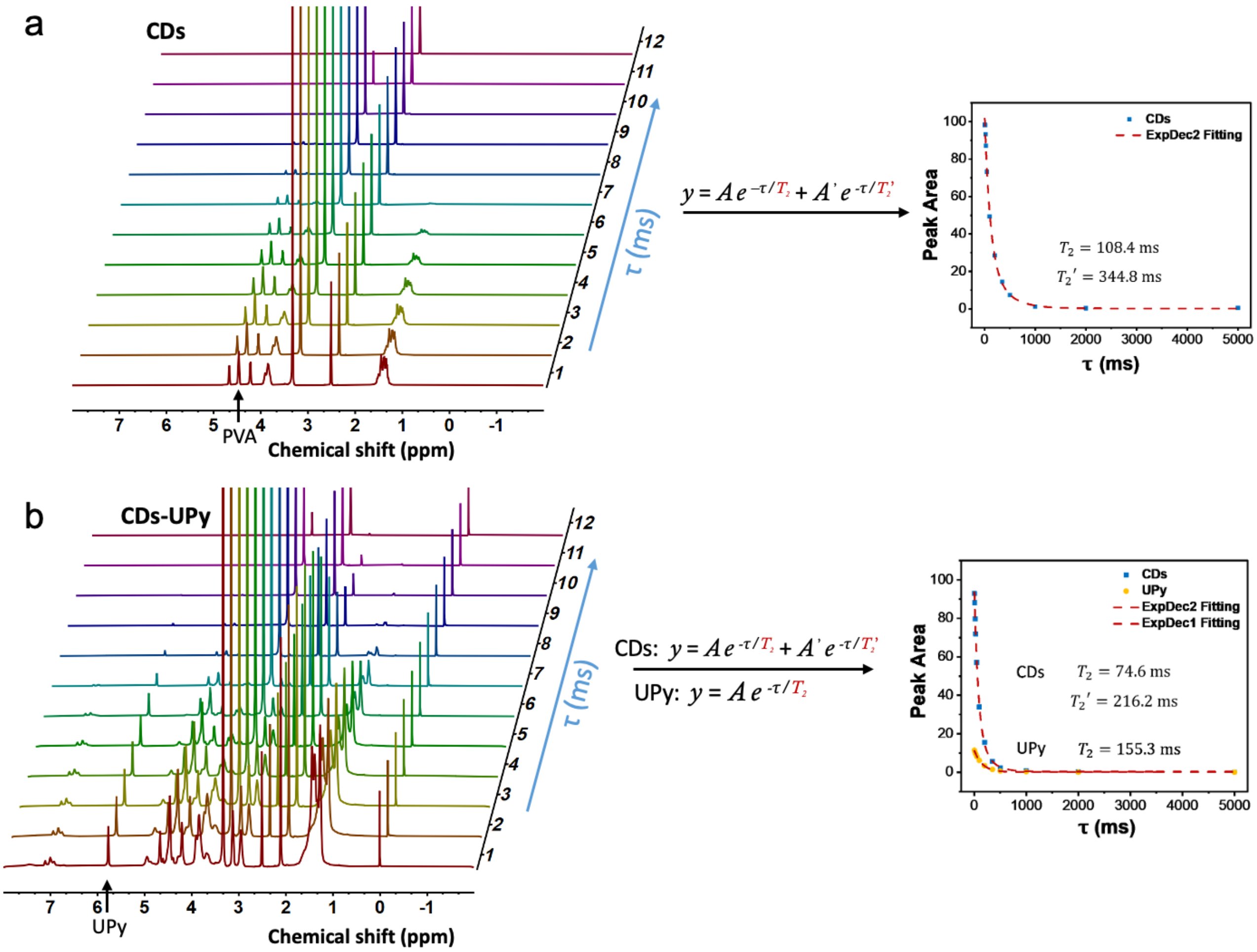

Supplementary Figure 8. Transverse relaxation times in NMR measurements. The CDs (a) and CDs-1.51UPy (b) fitting curves of the transverse relaxation peak area with respect to recovery time (τ). Source data are provided as a Source Data file.

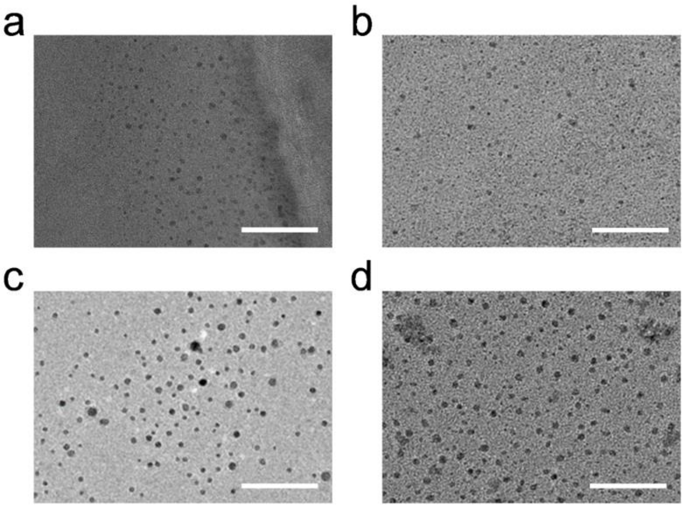

Supplementary Figure 9. TEM characterization of CDs-UPy. TEM images of CDs-0.89UPy (a), CDs-1.26UPy (b), CDs-1.51UPy (c), and CDs-1.80UPy (d). The scale bars are 50 nm.

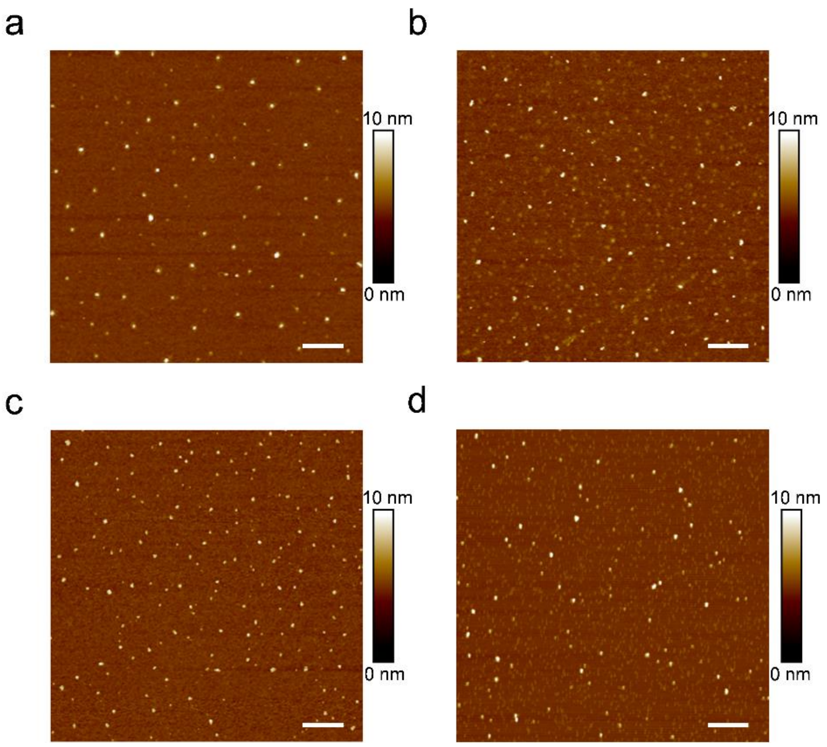

Supplementary Figure 10. AFM characterization of CDs-UPy. AFM images of CDs-0.89UPy (a), CDs-1.26UPy (b), CDs-1.51UPy (c), and CDs-1.80UPy (d). The scale bars are 500 nm.

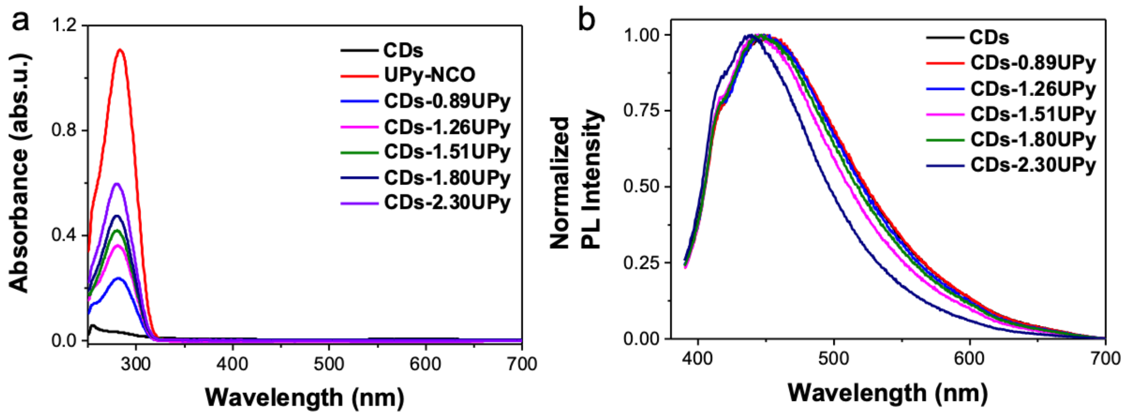

Supplementary Figure 11. Characterization of UV/Vis absorption spectra and fluorescence. (a) The UV/Vis absorption spectra of the CDs, UPy-NCO, and the CDs-UPy with different α values in DMSO. (b) The normalized PL spectra of the CDs and the CDs-UPy with different α values in DMSO. Source data are provided as a Source Data file.

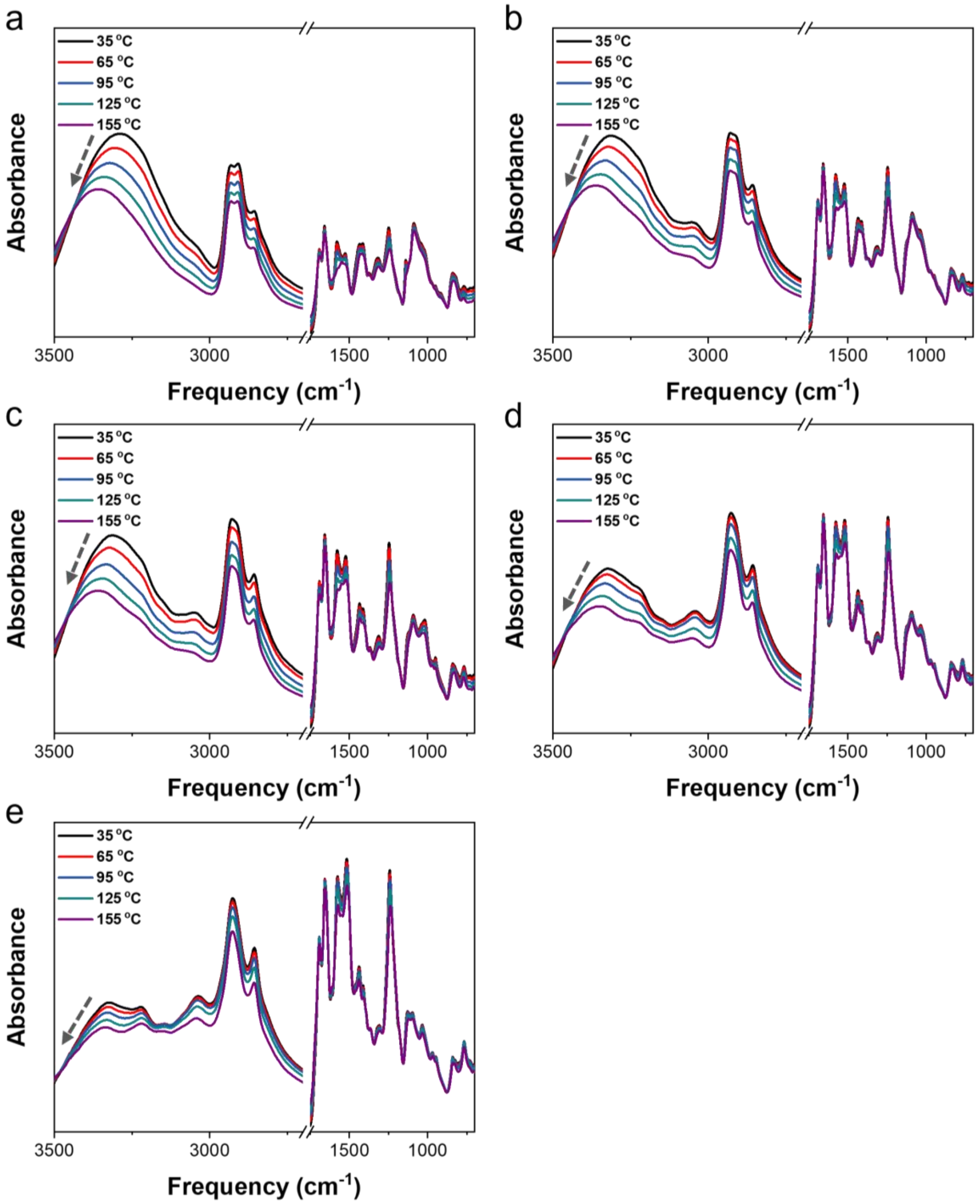

Supplementary Figure 12. FTIR characterization of CDs-UPy films at varied temperature. FTIR spectra of the CDs-UPy films with α of 0.89 (a), 1.26 (b), 1.51 (c), 1.80 (d), and 2.30 (e) mmol g^{-1} with the temperature changed in the range from 35 to 155 ^{o}C. Source data are provided as a Source Data file.

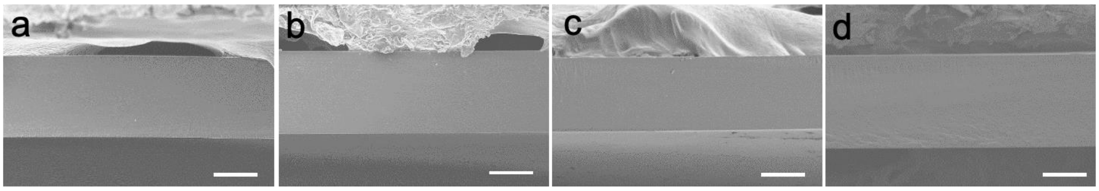

Supplementary Figure 13. SEM characterization of CDs-UPy films. Cross-sectional SEM images of the CDs-UPy macroscopic films with α of 0.89 (a), 1.26 (b), 1.51 (c), and 2.30 (d) mmol g^{-1}. The scale bars are 50 μm.

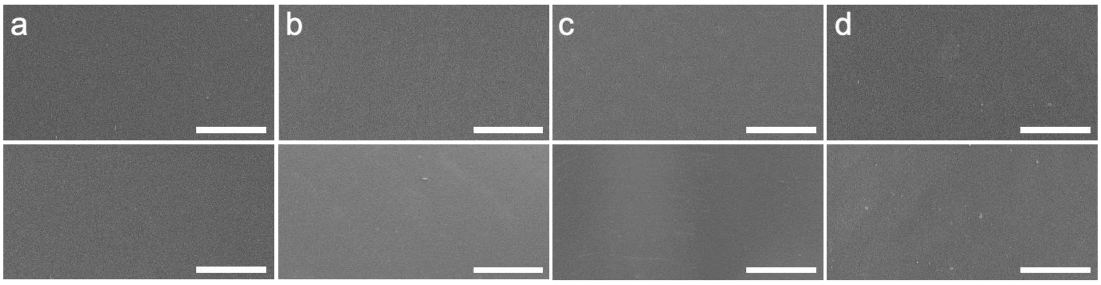

Supplementary Figure 14. SEM characterization of CDs-UPy films. SEM images of the upper surface (up) and lower surface (down) of the CDs-UPy macroscopic films with α of 0.89 (a), 1.26 (b), 1.51 (c), and 2.30 (d) mmol g^{-1}. The scale bars are 500 μm.

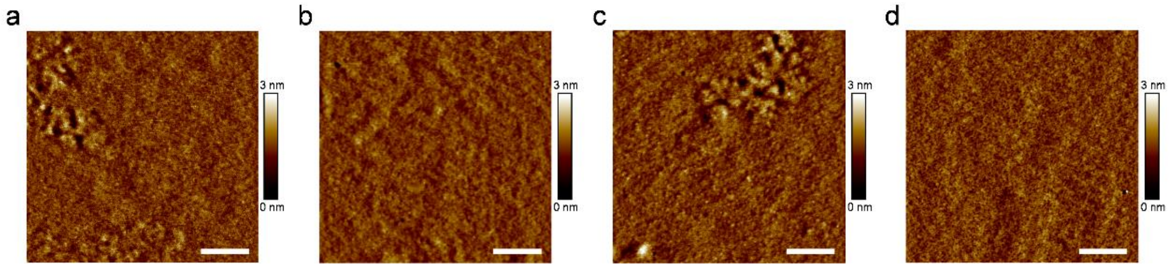

Supplementary Figure 15. AFM characterization of CDs-UPy films. AFM images of the CDs-UPy macroscopic films with α of 1.26 (a), 1.51 (b), 1.80 (c), and 2.30 (d) mmol g^{-1}. The scale bars are 400 nm. As α increases from 1.26 to 2.30 mmol g^{-1}, Ra and Rq are 0.34 and 0.46, 0.40 and 0.62, 0.24 and 0.32, 0.23 and 0.29 nm, respectively.

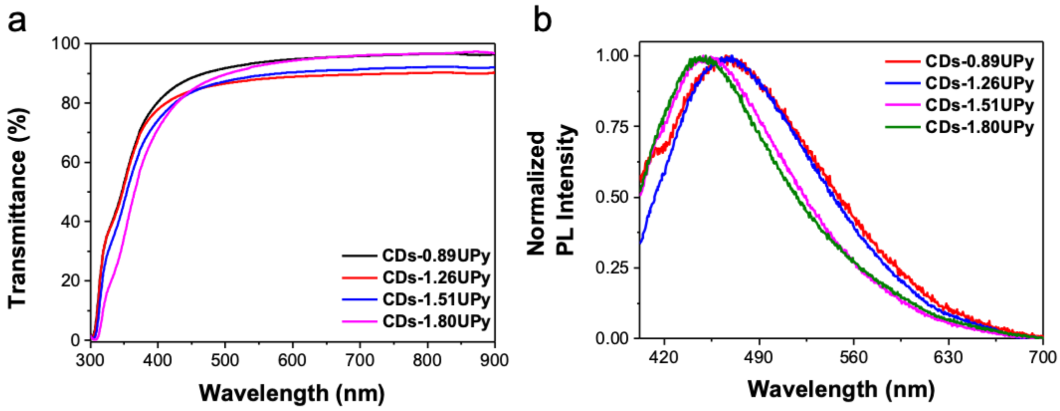

Supplementary Figure 16. Characterization of transmission spectra and fluorescence of the CDs-UPy films. (a) The transmission spectra of the CDs-UPy macroscopic films with different α values. (b) The normalized PL spectra of the CDs-UPy macroscopic films with different *α* values. Source data are provided as a Source Data file.

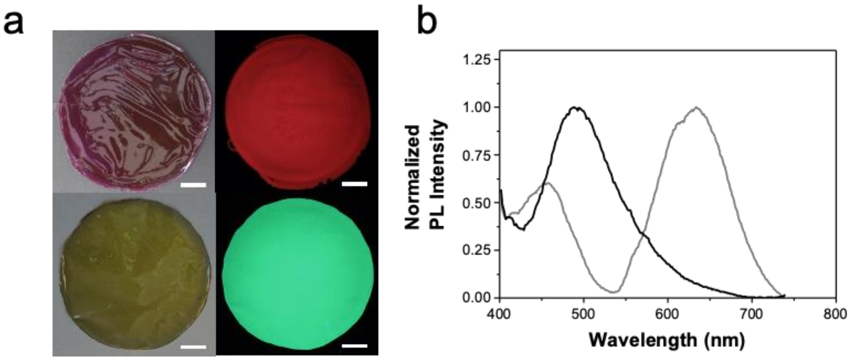

Supplementary Figure 17. Characterization of CDs-UPy films doped with red- and green-emissive CDs. (a) Representative photographs of the CDs-1.80UPy macroscopic films doped with red- (up) and green- (down) emissive CDs. The photographs were taken under the ambient light (left) and the ultraviolet lamp (right). The scale bars are 1 cm. (b) The normalized PL spectra of the CDs-1.80UPy macroscopic films doped with red- (gray line) or green-emissive (black line) CDs. Source data are provided as a Source Data file.

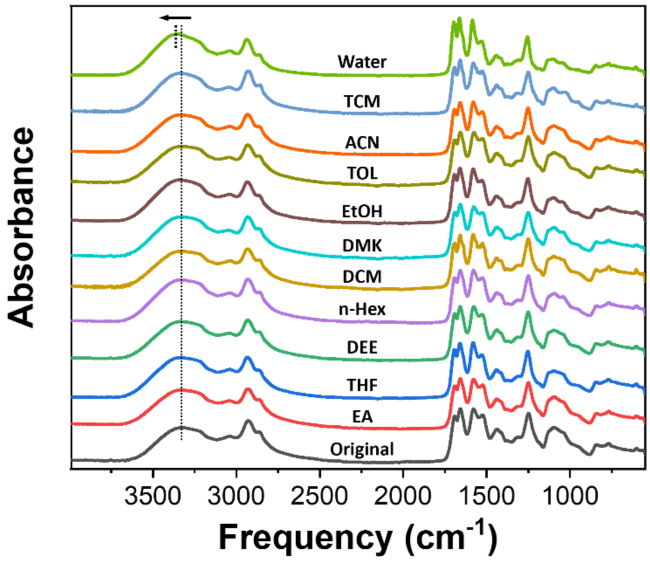

Supplementary Figure 18. FTIR characterization of CDs-UPy films upon the exposure of different solvents. FTIR spectra of the CDs-1.80UPy films before and after exposure to different solvents. The O-H or N-H stretching vibration of the CDs-1.80UPy films showed no shift after exposure to ten kinds of commonly used organic solvents. Whereas, the O-H or N-H stretching vibration of the CDs-UPy films shifted toward a higher frequency (a larger wavenumber) after exposure to water. The peak shift into large wavenumber indicated the partial breaking of hydrogen bonding among the CDs-UPy films after exposure to water. Ethyl acetate (EA), tetrahydrofuran (THF), diethyl ether (DEE), n-hexane (n-Hex), dichloromethane (DCM), acetone (DMK), ethanol (EtOH), toluene (TOL), acetonitrile (ACN), trichloromethane (TCM), and DI water (Water). Source data are provided as a Source Data file.

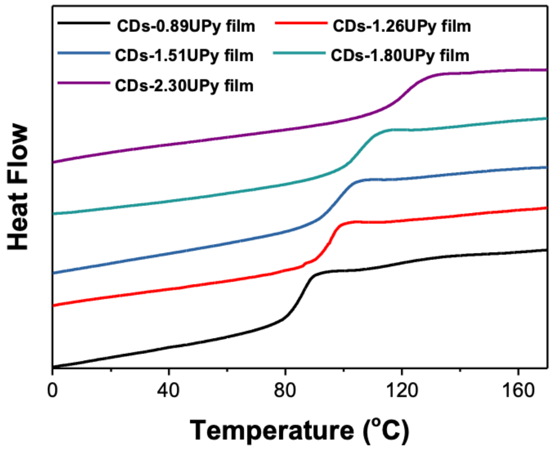

Supplementary Figure 19. DSC characterization of CDs-UPy films. DSC curves of the CDs-UPy films with α varying from 0.89 to 2.30. Source data are provided as a Source Data file.

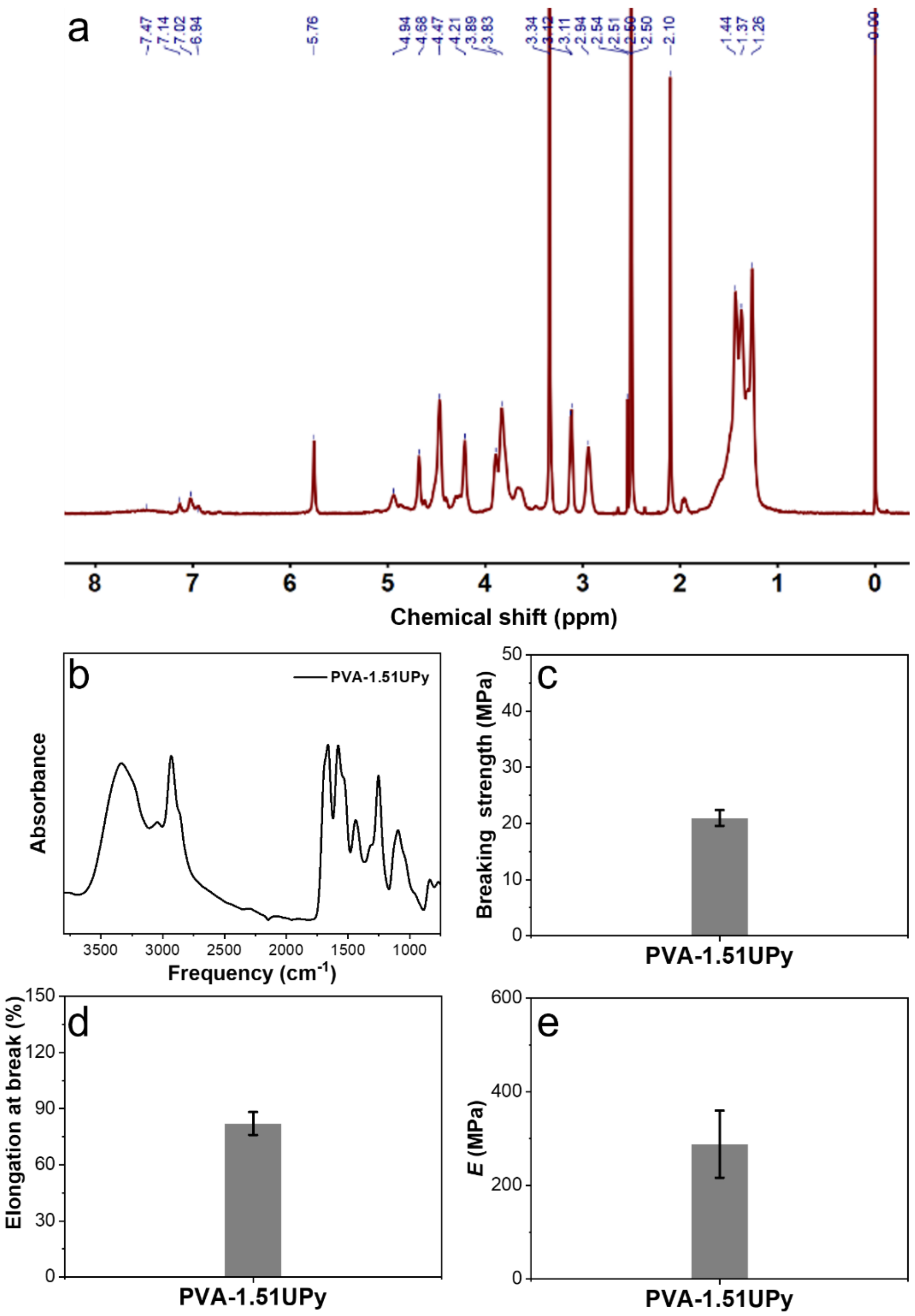

Supplementary Figure 20. Characterization of PVA-1.51UPy and PVA-1.51UPy films. (a) ^{1}H NMR spectrum of PVA-1.51UPy with DMSO-*d*$_6$ as solvent. (b) FTIR spectra of the PVA-1.51UPy. Breaking strength (c), elongation at break (d), and *E* (e) of PVA-1.51UPy films. The error bars represent standard deviations based on at least three independent measurements. Source data are provided as a Source Data file.

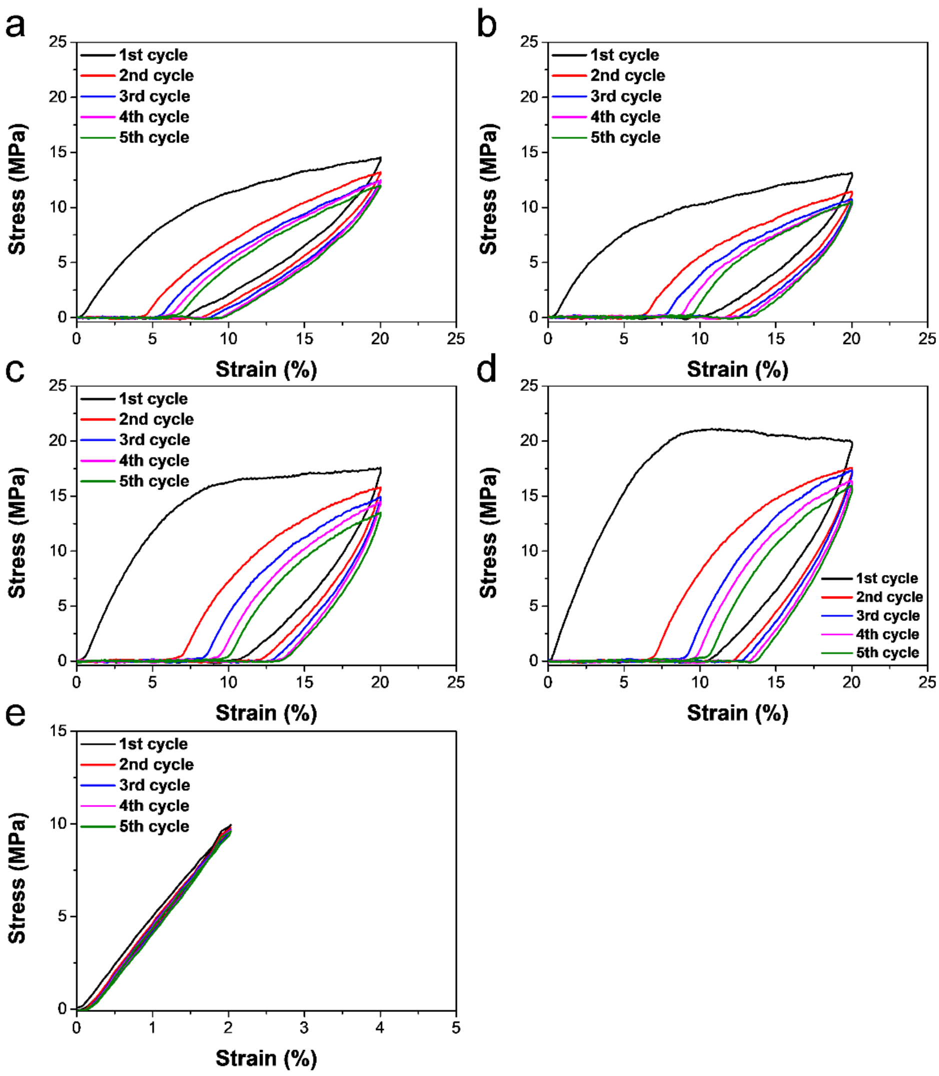

Supplementary Figure 21. Cyclic tensile loading–unloading tests of the CDs-UPy films. Stress−strain curves of five successive tensile loading-unloading cycles of the films assembled from CDs-0.89UPy (a), CDs-1.26UPy (b), CDs-1.51UPy (c), CDs-1.80UPy (d) and CDs-2.30UPy (e). Source data are provided as a Source Data file.

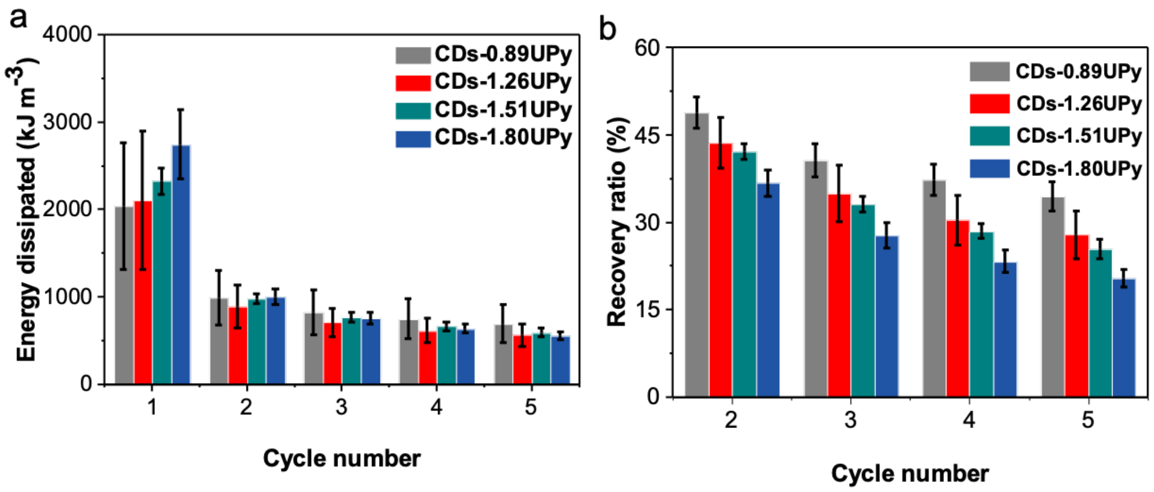

Supplementary Figure 22. Characterization of energy dissipation and recovery ratio. (a) Energy dissipation and recovery ratio (b) of CDs-UPy films with different α values upon cyclic loading-unloading tensile tests. The error bars represent standard deviations based on at least three independent measurements. Source data are provided as a Source Data file.

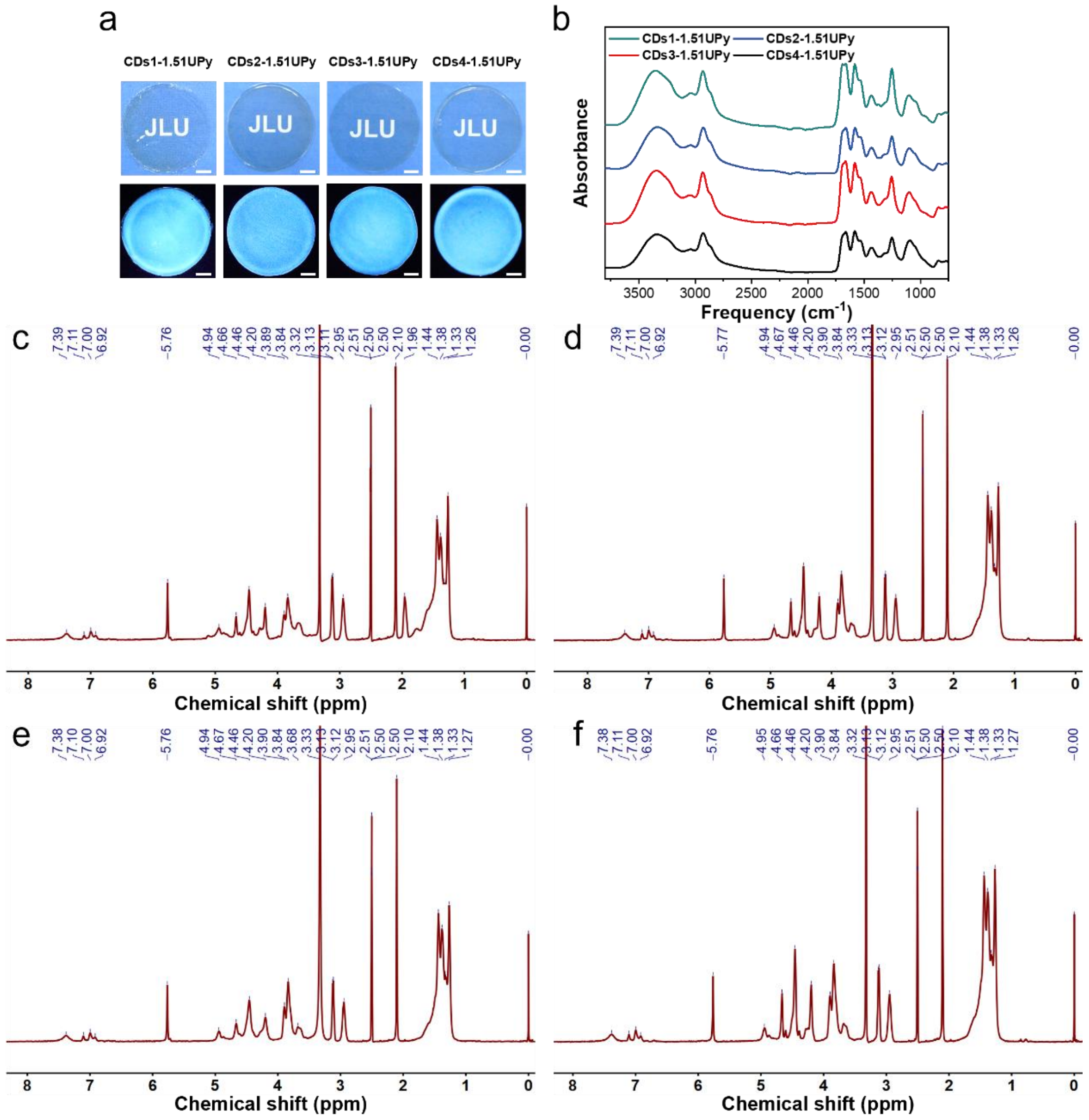

Supplementary Figure 23. Characterization of macroscopic films and CDs-UPy from different molecular weights. (a) Representative photographs of macroscopic films of the CDs1-1.51UPy, CDs2-1.51UPy, CDs3-1.51UPy, and CDs4-1.51UPy. The photographs were taken under the ambient light (up) and the ultraviolet lamp (down). The scale bars are 1 cm. (b) FTIR spectra of the CDs1-1.51UPy, CDs2-1.51UPy, CDs3-1.51UPy, and CDs4-1.51UPy. ^{1}H NMR spectrum of CDs1-1.51UPy (c), CDs2-1.51UPy (d), CDs3-1.51UPy (e), and CDs4-1.51UPy (f) with DMSO-d_6 as solvent. The CDs1, CDs2, CDs3, and CDs4 represent the CDs made from the molecular weights of polyvinyl alcohol as 9,000-10,000, 31,000-50,000, 89,000-98,000, and 130,000 g mol^{-1}, respectively. Source data are provided as a Source Data file.

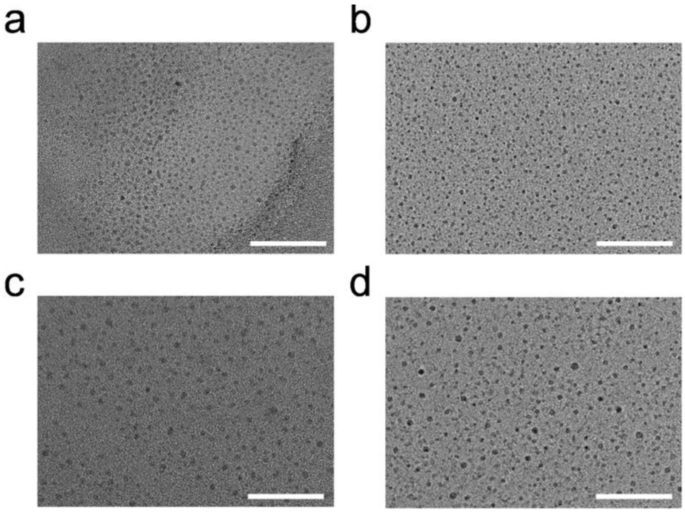

Supplementary Figure 24. TEM characterization of CDs-UPy from different molecular weights. TEM images of CDs1-1.51UPy (a), CDs2-1.51UPy (b), CDs3-1.51UPy (c), and CDs4-1.51UPy (d). The scale bars are 50 nm. The CDs1, CDs2, CDs3, and CDs4 represent the CDs made from the molecular weights of polyvinyl alcohol as 9,000-10,000, 31,000-50,000, 89,000-98,000, and 130,000 g mol^{-1}, respectively.

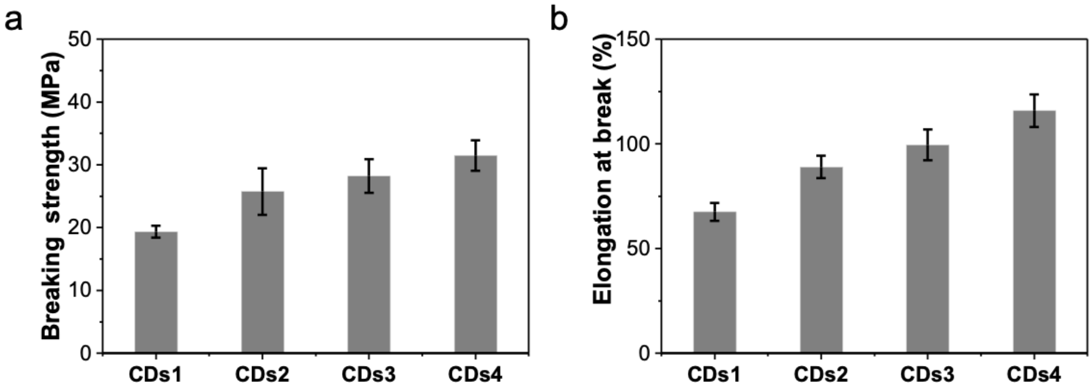

Supplementary Figure 25. The mechanical properties of CDs-UPy films from CDs-UPy of different molecular weights. Breaking strength (a) and elongation at break (b) of CDs-UPy films made from polyvinyl alcohol of different molecular weights with α of 1.51 mmol g^{-1}. The CDs1, CDs2, CDs3, and CDs4 represent the CDs made from the molecular weights of polyvinyl alcohol as 9,000-10,000, 31,000-50,000, 89,000-98,000, and 130,000 g mol^{-1}, respectively. The error bars represent standard deviations based on at least three independent measurements. Source data are provided as a Source Data file.

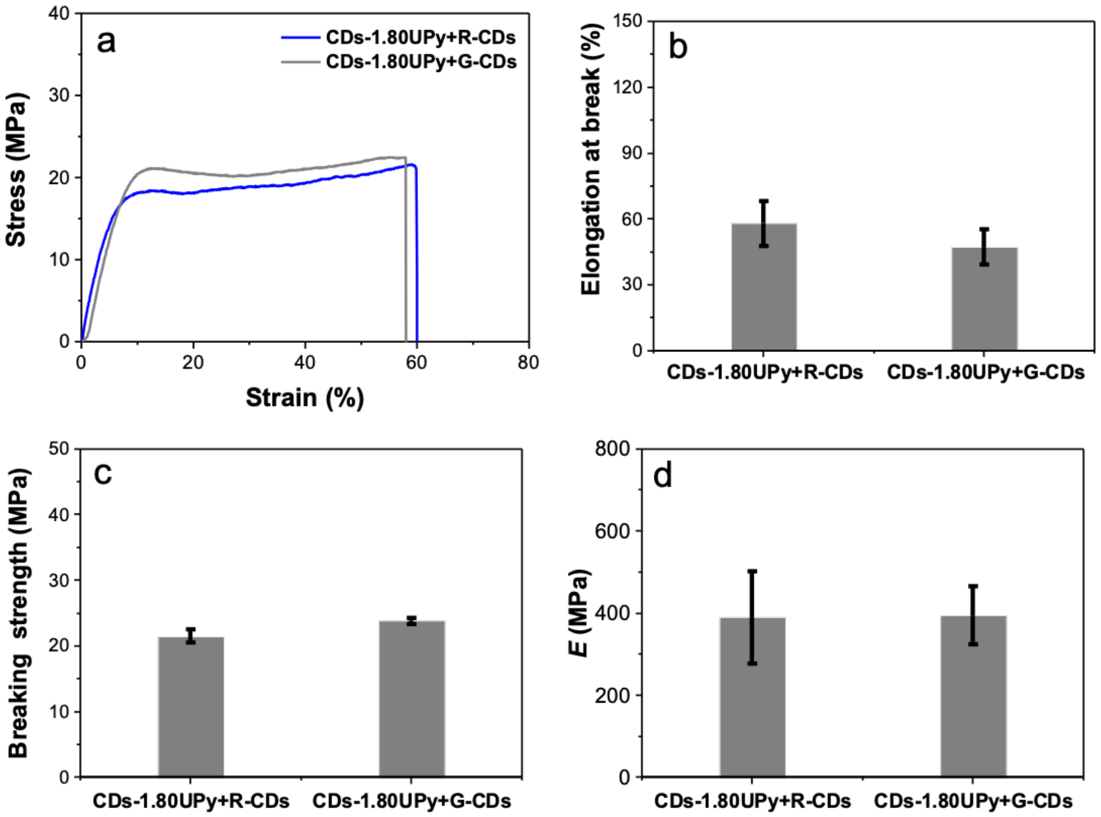

Supplementary Figure 26. The mechanical properties of CDs-UPy films doped with red- and green-emissive CDs. Stress−strain curves (a), elongation at break (b), breaking strength (c), and tensile Young's modulus, *E* (d) of CDs-1.80UPy films doped with red- or green-emissive CDs. G-CDs represents green-emissive CDs. R-CDs represents red-emissive CDs. The error bars represent standard deviations based on at least three independent measurements. Source data are provided as a Source Data file.

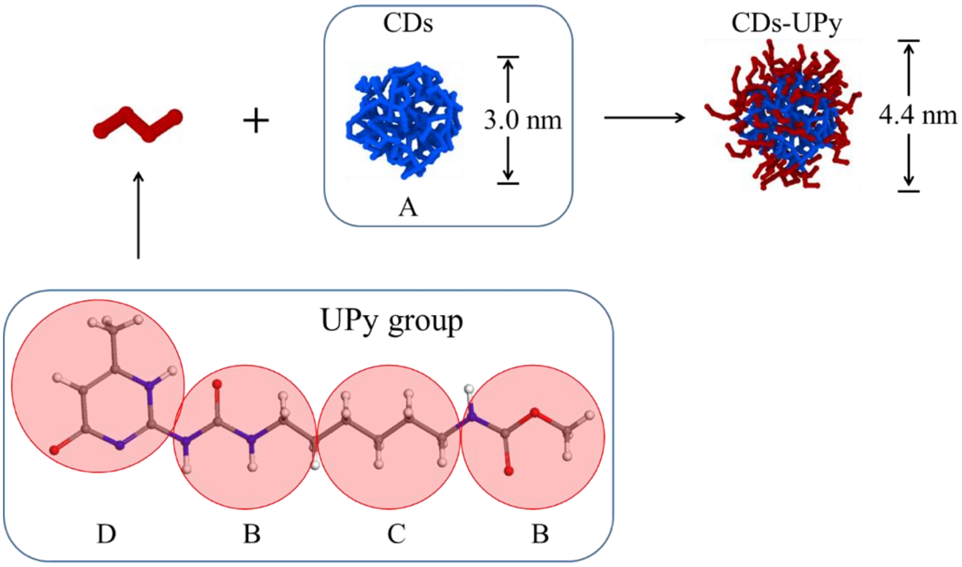

Supplementary Figure 27. The scheme of coarse-grained (CG) simulations. The CG scheme of UPy, CDs, and UPy- CDs in which the types of CG beads are labelled.

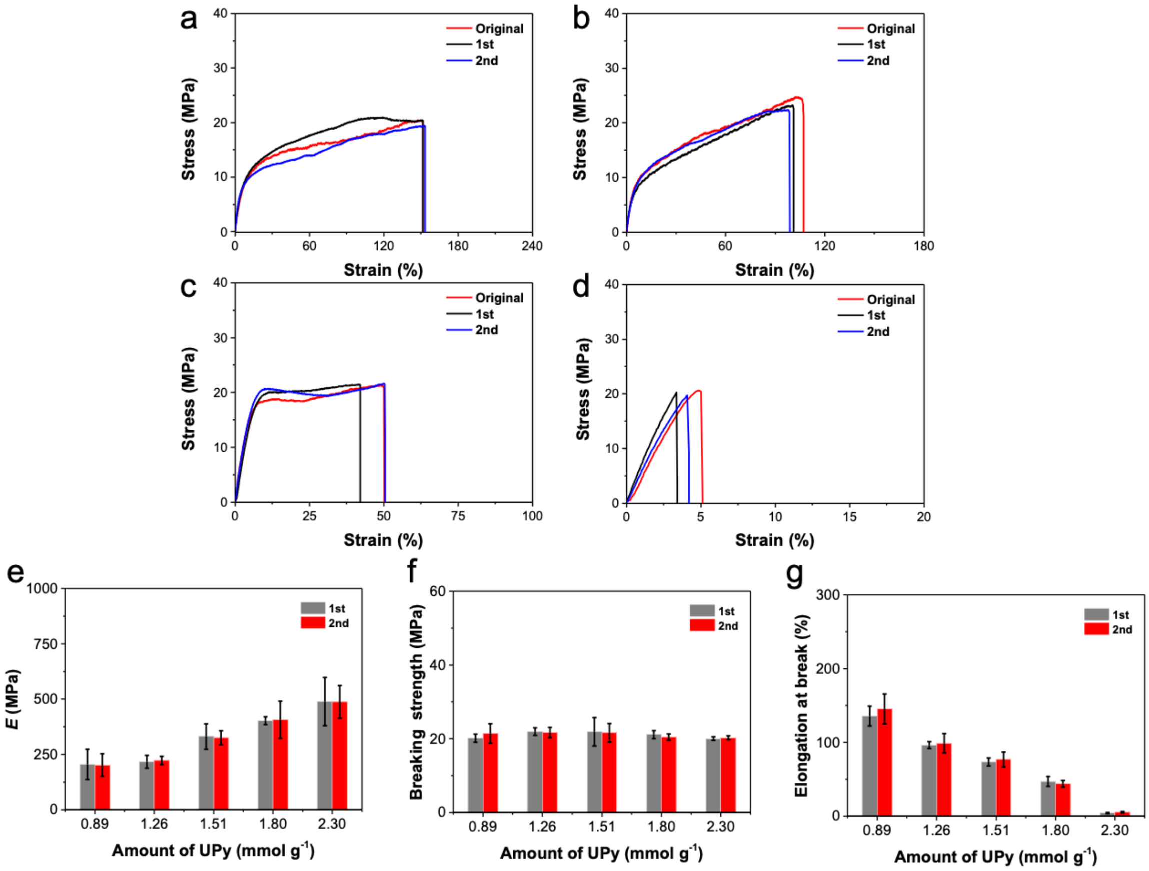

Supplementary Figure 28. The mechanical properties of CDs-UPy films after recasting. Stress−strain curves of CDs-UPy films with α of 0.89 (a), 1.26 (b), 1.80 (c), and 2.30 (d) mmol g^{-1} before and after recasting. Tensile Young's modulus, *E* (e), breaking strength (f), and elongation at break (g) of CDs-UPy films after recasting. The error bars represent standard deviations based on at least three independent measurements. Source data are provided as a Source Data file.

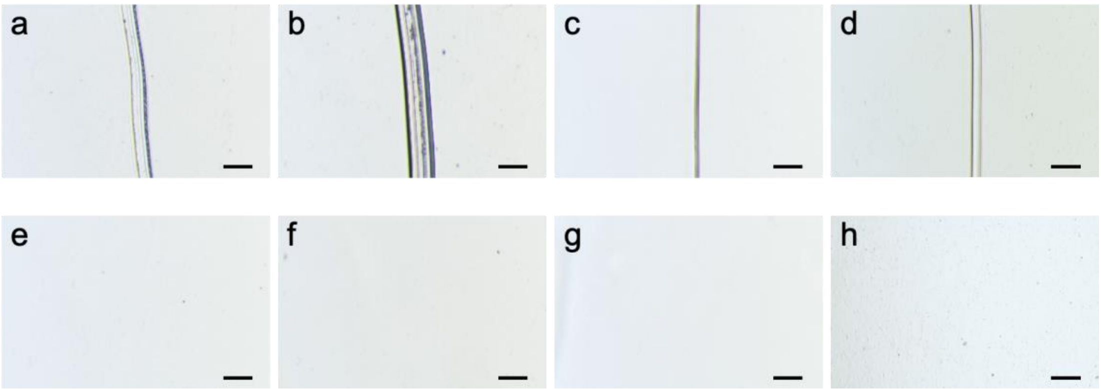

Supplementary Figure 29. The self-healing of CDs-UPy films. The photograph of scratch on the CDs-UPy films with α of 0.89 (a), 1.26 (b), 1.80 (c), and 2.30 (d) mmol g^{-1}. The photograph of the healed scratch on the CDs-UPy films with α of 0.89 (e), 1.26 (f), 1.80 (g), and 2.30 (h) mmol g^{-1}. The scale bars are 200 μm.

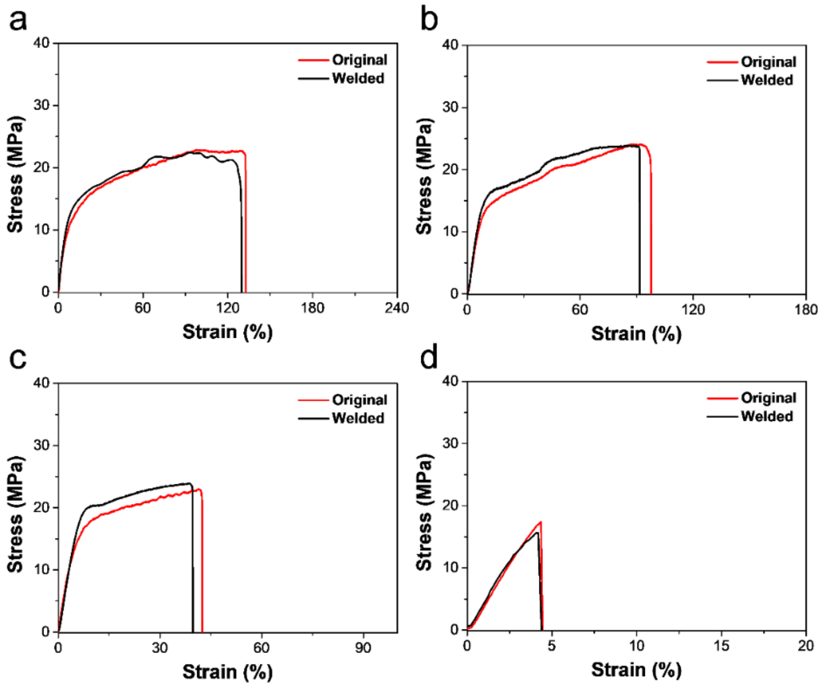

Supplementary Figure 30. The mechanical properties of CDs-UPy films after welding. Stress−strain curves of CDs-UPy films with α of 0.89 (a), 1.26 (b), 1.80 (c), and 2.30 (d) mmol g^{-1} before and after welding. Source data are provided as a Source Data file.

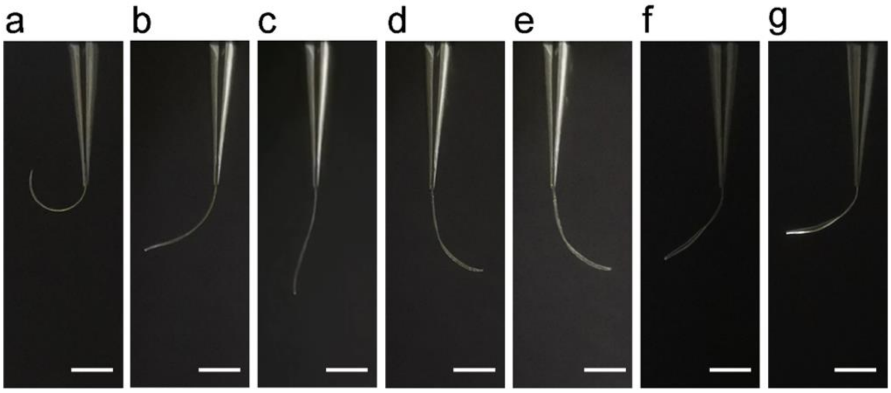

Supplementary Figure 31. The actuation of CDs-UPy films. Representative images of the double layer CDs-UPy film's bending in 100% humidity at 0 min (a), 10 min (b), 17 min (c), 20 min (d), 30 min (e), and then back in air with 30% humidity at 10 min (f), and 5 h (g). The double layer CDs-UPy film was composed of the CDs-0.89UPy films (left) and the CDs-1.80UPy films (right) by welding simply. The scale bars are 0.5 cm.

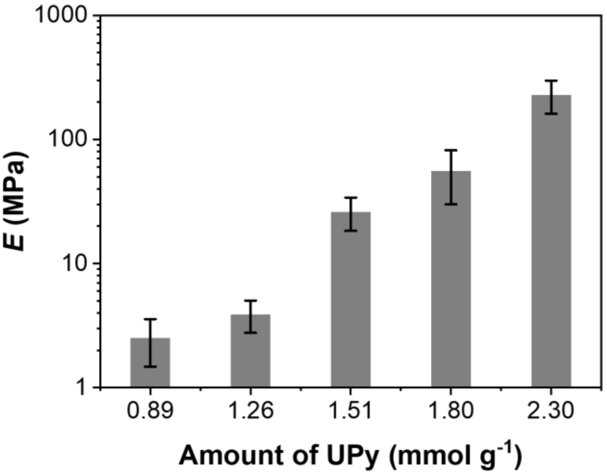

Supplementary Figure 32. The mechanical properties of CDs-UPy films after absorbing water. The tensile Young's modulus, *E* of CDs-UPy films with different α values in the wet state. The error bars represent standard deviations based on at least three independent measurements. Source data are provided as a Source Data file.

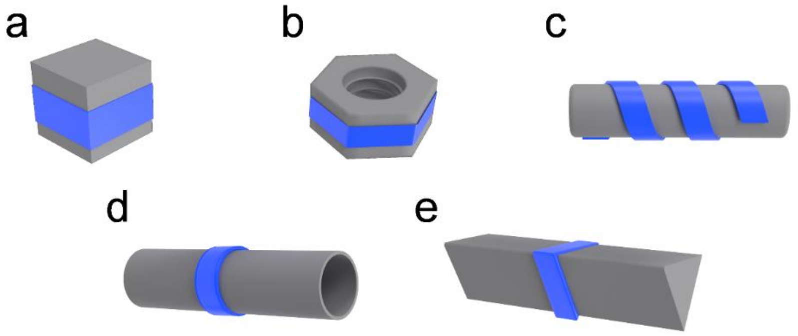

Supplementary Figure 33. Conceptual illustration diagrams of diverse programmed shaping of CDs-UPy film stripes. (a-e) The schematic of showing a square (a), a hexagon (b), a helix (c), a ring (d), and a triangle (e) on corresponding moulds.

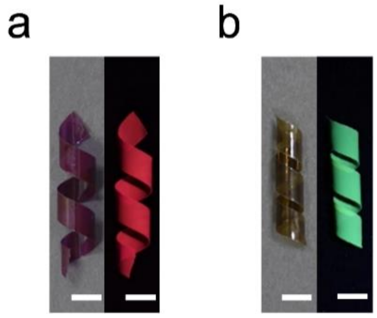

Supplementary Figure 34. Shape-programming of CDs-UPy films doped with red- or green-emissive CDs. Representative photographs of the helix programmed from the CDs-1.80UPy films doped with red- (a) or green-emissive CDs (b). The scale bars are 0.5 cm. The photographs were taken under the ambient light (left) and the ultraviolet lamp (right).

Supplementary Tables

Supplementary Table 1. Elemental analysis of the CDs, CDs-UPy with different α values, PVA-1.51UPy, and the CDs-UPy made from different molecular weights of polyvinyl alcohol with α of 1.51 mmol g^{-1}.

	**N (%)**	**C (%)**	**H (%)**
**CDs**	0.00	53.20	9.131
**CDs-0.89UPy**	6.23	51.12	8.397
**CDs-1.26UPy**	8.85	52.91	8.195
**CDs-1.51UPy**	10.56	52.56	8.014
**CDs-1.80UPy**	12.57	52.92	7.389
**CDs-2.30UPy**	16.09	52.51	7.389
**PVA-1.51UPy**	10.50	52.16	8.010
**CDs1-1.51UPy**	10.38	54.85	7.860
**CDs2-1.51UPy**	10.53	51.56	7.789
**CDs3-1.51UPy**	10.69	51.59	7.819
**CDs4-1.51UPy**	10.59	52.21	7.944

Supplementary Table 2. The mass formula of the CDs and UPy-NCO for the preparation of CDs-UPy with different α values.

	**CDs (g)**	**UPy-NCO (g)**
**CDs-0.89UPy**	2.20	0.75
**CDs-1.26UPy**	2.20	1.50
**CDs-1.51UPy**	2.20	2.00
**CDs-1.80UPy**	2.20	3.00
**CDs-2.30UPy**	2.20	6.00

Supplementary Table 3. The comparison of the mechanical properties of the CDs-UPy films with those of macroscopic materials assembled from other nanoparticles

Samples	*E* (MPa)	Breaking strength (MPa)	Elongation at break (%)	Ref.
CDs-0.89UPy	≈ 196	≈ 20	≈ 138	This work
CDs-1.26UPy	≈ 216	≈ 20	≈ 100	This work
CDs-1.51UPy	≈ 329	≈ 20	≈ 74	This work
CDs-1.80UPy	≈ 403	≈ 20	≈ 47	This work
CDs-2.30UPy	≈ 490	≈ 20	≈ 5	This work
CDs1-1.51UPy	≈ 300	≈ 19	≈ 68	This work
CDs2-1.51UPy	≈ 368	≈ 26	≈ 89	This work
CDs3-1.51UPy	≈ 425	≈ 28	≈ 100	This work
CDs4-1.51UPy	≈ 477	≈ 31	≈ 116	This work
Polymer-grafted silica nanoparticles	≈ 82	≈ 14	≈ 58	[1]
Metal–Organic Polyhedron Supra-Nanoparticle Clusters	≈ 17	≈ 1	≈ 15	[2]
Metal–Organic Polyhedron Nanocomposites	0.2	0.06	150	[3]
SiO2-PNIPAAm nanoparticles	NA	< 0.04	40	[4]
GSH-CdTe hydrogel-544	≈ 5/≈ 1.3 MPa (storage modulus/loss modulus)	NA	NA	[5]

*NA: not available

Supplementary Table 4. The parameters of non-bonded interactions.

$\epsilon_{ij}/\sigma_{ij}$	**A**	**B**	**C**	**D**
**A**	$3.5\epsilon_0/0.47\sigma_0$	$2.7\epsilon_0/0.47\sigma_0$	$3.5\epsilon_0/0.47\sigma_0$	$3.0\epsilon_0/0.51\sigma_0$
**B**		$4.0\epsilon_0/0.47\sigma_0$	$2.7\epsilon_0/0.47\sigma_0$	$5.0\epsilon_0/0.51\sigma_0$
**C**			$3.5\epsilon_0/0.47\sigma_0$	$3.0\epsilon_0/0.51\sigma_0$
**D**				$5.6\epsilon_0/0.55\sigma_0$

Supplementary Note 1. Discussion on the preparation of CDs and CDs-UPy

The pristine CDs was prepared through heating an aqueous solution of polyvinyl alcohol in a sealed Teflon-lined stainless-steel autoclave at 200 °C for 6 h. After the hydrothermal treatment, a very small quantity of leftover PVA was removed through the rigorous dialysis procedure. To remove leftover PVA (M_w, 13,000 g mol^{-1}), the CDs was dialyzed against DI water (4000 mL) through a dialysis membrane (MWCO 50 kDa) for 7 days and the DI water was changed three times every day. The formation of CDs has been proved by the TEM, AFM, and DLS which were usually used as effective tools to characterize nanoparticles (Supplementary Fig. 1a-d). Supplementary Fig. 1a shows the representative transmission electron microscopy (TEM) images of the pristine CDs. The pristine CDs shows a dot-like shape with an average diameter of 2.8 ± 0.5 nm. Supplementary Fig. 1b shows the high-resolution TEM images of the pristine CDs with an average lattice spacing of 0.21 nm corresponding to the d-spacing of the graphene (100) planes, which indicated that the CDs have a certain degree of crystallinity. Supplementary Fig. 1c shows the representative atomic force microscopy (AFM) images of the pristine CDs. The CDs showed ≈ 3 nm in height determined by AFM measurements, which were in agreement with their average diameter determined by TEM. Supplementary Fig. 1d shows the DLS data of CDs in DMSO and H_2O. Due to the swelling by water, the CDs showed a hydrodynamic diameter of ≈ 7 nm in water, which was larger than the results of TEM and AFM measurements. The above evidence clearly demonstrated the morphology and size of CDs. Supplementary Fig. 1e shows the Raman spectra of the pristine CDs. The appearances of D and G band in the Raman spectra of CDs indicated the successful preparation of CDs. Moreover, the CDs obtained after hydrothermal treatment showed significantly enhanced fluorescence intensity in comparison with the same mass concentration of PVA solution (Supplementary Fig. 1f). The significantly enhanced fluorescence derived from the characteristic structure of CDs, which further suggests the formation of CDs.

The core-shell structure of CDs and CDs-UPy was characterized by transverse (T_2) relaxation times in NMR measurements[6–8] (Supplementary Fig. 8). The pristine CDs prior to the UPy grafting showed two T_2 values. The smaller T_2 value, 108.4 ms, was derived from the core of the pristine CDs. The larger T_2 value, 344.8 ms, was resulted from the outside polymer chains (or branches) of the pristine CDs. After the modification of UPy motifs, CDs-UPy showed two T_2 values derived from the PVA motifs, indicating that the core-shell structure of the pristine CDs still remained after the UPy grafting. The UPy motifs of CDs-UPy only showed single T_2 value, 155.3 ms. Because of the steric hindrance, the UPy motifs of CDs-UPy could be only grafted on the shell of the pristine CDs. These results suggested that both CDs and CDs-UPy showed core-shell structure.

Supplementary Note 2. Actuating application of CDs-UPy films

Humidity-responsive actuators based on the CDs-UPy films were fabricated by the welding experiments. In brief, the CDs-0.89UPy films with a width of 1 cm and a length of 2 cm were overlapped with the same size of CDs-1.80UPy films, and then this double-layer sample was heated at 60 ^{o}C under the pressure of 500 g weight and the assistance of the trace amount of water. After 1 h, the CDs-0.89UPy films were integrated with the CDs-1.80UPy films by welding simply, thus forming a double layer material. The double layer materials were cut into strips with a width of 1 mm and a length of 1.5 cm for actuating application (Supplementary Fig. 31).

The different layers of the integrated materials showed different swelling behaviors in water (Supplementary Fig. 32). The differences in the absorption of water molecule will lead to the differences in volume between the double layers of the strip. The differences in volume could be regarded as the driving force of the actuation. By utilizing the differences of swelling ratios in water, the double-layer film could be used as actuators with fluorescence towards 100% humidity air. Initially, the double-layer strip bent towards the left side (the CDs-0.89UPy film). During the welding experiment, CDs-0.89UPy films swelled more than the CDs-1.80UPy films, because CDs-0.89UPy films absorbed more water than CDs-1.80UPy films (Supplementary

Fig. 32). After drying, CDs-0.89UPy side shrank more than that of CDs-1.80UPy films, leading to the bending towards the side of the CDs-0.89UPy film (Supplementary Fig. 31a). Upon the exposure in 100% humidity air, left side (the CDs-0.89UPy film) swelled more than the right side (the CDs-1.80UPy films) by water, which drove the double-layer film bending towards right side. The double-layer CDs-UPy film bent towards the right side of CDs-1.80UPy films gradually from 0 to 17 min (Supplementary Fig. 31a-c). The double-layer strip bent to a nearly straight film after the exposure in 100% humidity air for 17 min (Supplementary Fig. 31c). After that, the double-layer strip bent towards the right side (the CDs-1.80UPy films) gradually from 17 to 20 min. The double-layer strip bent towards the left side (the CDs-0.89UPy films) upon exposure in 30% humidity environment for 10 min (Supplementary Fig. 31f). Bending back to the left side was relatively slow (Supplementary Fig. 31g).

Supplementary references

1. Williams, G. A. *et al.* Mechanically Robust and Self-Healable Superlattice Nanocomposites by Self-Assembly of Single-Component ‘Sticky’ Polymer-Grafted Nanoparticles. *Adv. Mater.* **27**, 3934–3941 (2015).
2. Yin, J. F. *et al.* Unexpected Elasticity in Assemblies of Glassy Supra-Nanoparticle Clusters. *Angew. Chem. Int. Ed.* **60**, 4894–4900 (2021).
3. Zhang, M. *et al.* The Microscopic Structure–Property Relationship of Metal–Organic Polyhedron Nanocomposites. *Angew. Chem. Int. Ed.* **58**, 17412–17417 (2019).
4. Liu, M. *et al.* Thermo-Responsive Jamming of Nanoparticle Dense Suspensions towards Macroscopic Liquid–Solid Switchable Materials. *Angew. Chem. Int. Ed.* **61**, e202114602 (2022).
5. Zhou, Y. *et al.* Unusual multiscale mechanics of biomimetic nanoparticle hydrogels. *Nat. Commun.* **9**, 181 (2018).
6. Pietrasik, J., Sumerlin, B. S., Lee, H. il, Gil, R. R. & Matyjaszewski, K. Structural mobility of molecular bottle-brushes investigated by NMR relaxation dynamics. *Polymer* **48**, 496–501 (2007).
7. Wu, M. *et al.* Solution NMR Analysis of Ligand Environment in Quaternary Ammonium-Terminated Self-Assembled Monolayers on Gold Nanoparticles: The Effect of Surface Curvature and Ligand Structure. *J. Am. Chem. Soc.* **141**, 4316–4327 (2019).

8. Kim, Y. G., Wagner, M. & Thérien-Aubin, H. Dynamics of Soft and Hairy Polymer Nanoparticles in a Suspension by NMR Relaxation. *Macromolecules* **53**, 844–851 (2020).
