# Supplementary material for: Recastable assemblies of carbon dots into mechanically robust macroscopic materials
Source: Nat Commun. 2023 Oct 25;14:6782. doi: 10.1038/s41467-023-42516-8 (PMC10600192; doi:10.1038/s41467-023-42516-8)
Supplement: Supplementary file 2 — Description of Additional Supplementary Files [file 41467_2023_42516_MOESM2_ESM.pdf]

Description of Additional Supplementary Files

File Name: Supplementary Movie 1
Description: Structure evolution of the CDs-0.89UPy films during the elongation process.

File Name: Supplementary Movie 2
Description: Structure evolution of the CDs-1.80UPy films during the elongation process.
